# Global burden of epilepsy attributable to neonatal disorders in children from 1990 to 2021

**DOI:** 10.1016/j.isci.2026.115296

**Published:** 2026-03-10

**Authors:** Shanying Zhong, Yihe Lian, He Yi, Kaiyun Jia, Ningning Zhang, Patrick Kwan, Yong Yang, Xin Tian

**Affiliations:** 1 Department of Geriatrics, Laboratory of Research and Translation for Geriatric Diseases, The First Affiliated Hospital of Chongqing Medical University, Chongqing 400016, China; 2 Department of Neurology, The First Affiliated Hospital of Chongqing Medical University, Chongqing Key Laboratory of Major Neurological and Mental Disorders, Chongqing 400016, China; 3 Department of Epilepsy Center, The First Affiliated Hospital of Chongqing Medical University, Chongqing 400016, China; 4 Key Laboratory of Major Brain Disease and Aging Research (Ministry of Education), Chongqing Medical University, Chongqing 400016, China; 5 The Second Hospital and Clinical Medical School, Lanzhou University, Lanzhou 730030, China; 6 Department of Neuroscience, Central Clinical School, Monash University, Melbourne, VIC 3004, Australia; 7 Department of Neurology, The Affiliated Hospital of Qingdao University, Qingdao 266003, China

**Keywords:** health sciences, medical specialty, medicine, neurology, public health

## Abstract

This study provides a comprehensive assessment of the global burden of childhood epilepsy attributable to neonatal disorders, using data from the Global Burden of Disease Study 2021. From 1990 to 2021, the age-standardized prevalence rate and number of years lived with disability increased globally, with higher burdens observed among boys and in regions with lower Sociodemographic Index levels. Preterm birth was identified as the most significant neonatal cause of childhood epilepsy. These findings depict the burden of epilepsy attributable to neonatal disorders in children and underscore the need for targeted perinatal care strategies, particularly in low-resource settings, to reduce the long-term neurological sequelae in children worldwide.

## Highlights

• There was an ascending trend during 1990–2021
• The ASPR and YLDs were higher in boys than in girls
• The burden was more notable in regions with lower SDI
• Preterm birth was the most common cause

## Graphical abstract

**Figure:**
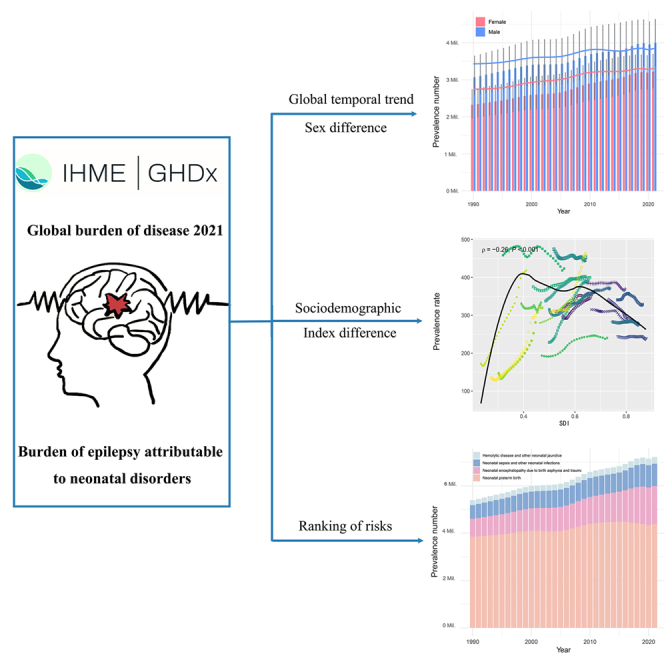

## Introduction

The burden of epilepsy, one of the most common serious brain conditions affecting individuals of all ages across the globe, has been increasing. Epilepsy increases the risk of premature death 3-fold compared with the general population. A capstone publication of the Global Burden of Disease (GBD) 2021 about epilepsy reported that there were 51.7 million people with epilepsy in 2021, with a prevalence rate of 0.7%. Among those individuals, more than 50% had secondary epilepsy, which is difficult to effectively control with antiepileptic drugs.^1^ However, secondary epilepsy is often preventable through targeted etiological interventions. As an important public health issue, epilepsy has already attracted attention from the global community. In 2022, the World Health Organization (WHO) adopted the Intersectoral Global Action Plan on Epilepsy and Other Neurological Disorders (2022–2031). This action plan highlights the importance of preventing the cause of the disease through various methods to alleviate the burden of secondary epilepsy.^2^

Neonatal disorders refer to a category of diseases that originate during the prenatal or perinatal period and manifest major symptoms during the neonatal period. Previous studies have demonstrated that many neonatal disorders can cause epilepsy. For instance, a multicenter study reported that preterm infants face an increased risk later in childhood compared with infants born at term, with a hazard ratio of 3.7.^3^ A nationwide cohort study reported that neonatal sepsis is associated with an approximately 3-fold increased risk of childhood epilepsy.^4^ Epilepsy attributable to neonatal disorders refers to epilepsy caused by brain damage resulting from neonatal conditions, which is in accordance with the International Classification of Diseases 11 (ICD-11) code: 8A60.0.^5^

Epilepsy attributable to neonatal disorders imposes a substantial burden on societies and affects both children and their families. The most effective strategy for reducing the incidence of epilepsy linked to neonatal disorders is to provide high-quality care during the perinatal period. However, due to underdeveloped medical situations, some areas face a higher burden of neonatal disorders, resulting in a heavy burden of secondary epilepsy. The Sustainable Development Goals (SDGs) aim to reduce premature mortality from non-communicable diseases by one-third and eliminate preventable deaths of newborns.^2^^,^^6^ Consequently, it is essential to obtain global epidemiological data for developing effective public health strategies to alleviate the burden of childhood epilepsy attributable to neonatal disorders.

The GBD 2021 study offers a comprehensive evaluation of the burdens of epilepsy and types of injuries across 204 countries and territories since 1990.^7^ A previous study based on GBD 2021 reported that the prevalence of childhood epilepsy was 6.10 million, with a prevalence rate of 301.4 per 100,000 in 2021. Childhood epilepsy also accounted for 18,171 deaths and 3.56 million disability-adjusted life years.^8^ Over the past 30 years, the prevalence of childhood epilepsy has increased.^9^ Boys and newborns under the age of 1 year are more susceptible to epilepsy. Children with epilepsy living in low Sociodemographic Index regions have a higher risk of mortality.^10^ Although the burden of childhood epilepsy has previously been demonstrated, epilepsy specifically attributable to neonatal disorders and its spatiotemporal distribution pattern have not been depicted. Our study aims to expound a global assessment of childhood epilepsy attributable to neonatal disorders and depicts the disparity of the disease burden in populations stratified by sex, age group, SDI, region, and nation. Our research can significantly assist healthcare professionals in developing innovative prevention and treatment strategies to mitigate the health risks associated with childhood epilepsy.

## Results

### Temporal trends in the global burden

**Figure 1:**
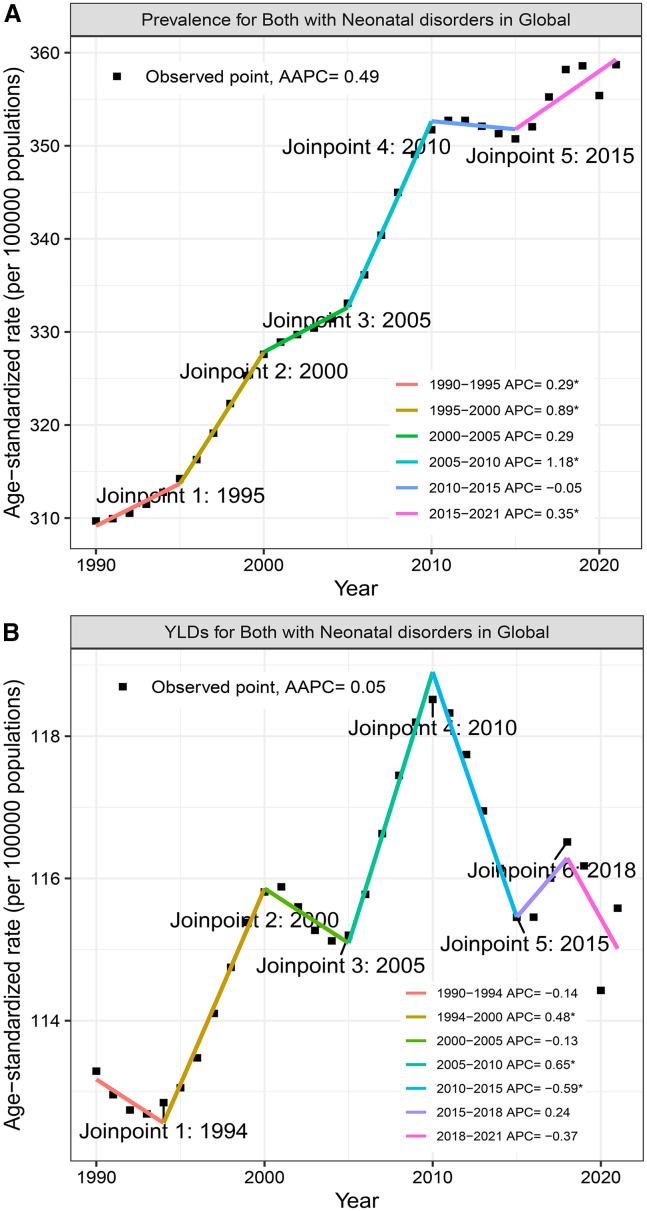
Global temporal trends for age-standardized rates from 1990 to 2021 (A) Age-standardized prevalence rate (ASPR). (B) Age-standardized YLD rate. Abbreviations: APC = annual percent change, AAPC = average annual percent change, YLDs = years lived with disability. The estimated value is represented by the median of the data distribution.

In 2021, there were 7.2 million (95% uncertainty intervals (UIs): 6.3 to 8.1) childhood epilepsy cases globally attributed to neonatal disorders in children, with an age-standardized prevalence rate (ASPR) of 358.7 (95% UI: 306 to 413.5) per 100,000 (Table 1). From 1990 to 2021, the ASPR showed an increasing trend, rising from 309.7 (95% UI: 262.6 to 365.6) per 100,000 to 358.7 (95% UI: 306 to413.5) per 100,000, with an estimated annual percentage change (EAPC) of 0.54 (95% confidence interval (CI), 0.50 to 0.58) (Table 1). Notably, the ASPR sharply increased from 1995 to 2010. The ASPR rose from 314.1 in 1995 to 352.5 in 2010 per 100,000, increasing by 38.4 per 100,000. The period of 1995–2010 accounted for 78% of the total increase in the past 30 years. This trend was visualized using the joinpoint model (Figure 1).

**Table 1:** Age-standardized prevalence and EAPC of epilepsy caused by neonatal disorders in children under 15 years, global and regional level, 1990-2021

	1990		2021		1990–2021
Characters	Prevalent cases (thousands) (95% UI)	ASPR (95% UI)	Prevalent cases (thousands) (95% UI)	ASPR (95% UI)	EAPC (95% CI)
Global	5392.6 (4646.8–6313.6)	309.7 (262.6–365.6)	7218.2 (6296.3–8113)	358.7 (306–413.5)	0.54 (0.5–0.58)

**Sex**					

Male	3068.6 (2635.1–3582.2)	343 (289.9–407.4)	3996.8 (3460.3–4509.1)	384.7 (324.8–445.6)	0.42 (0.38–0.46)
Female	2324 (2002.9–2704.5)	274.5 (232.6–322.8)	3221.4 (2848.2–3593.4)	331 (285.5–379.1)	0.69 (0.65–0.73)
Both	5392.6 (4646.8–6313.6)	309.7 (262.6–365.6)	7218.2 (6296.3–8113)	358.7 (306–413.5)	0.54 (0.5–0.58)

**Age**					

0-4 years	2127.4 (1823.9–2505.1)	343.2 (294.2–404.1)	2347.1 (2012.1–2719.1)	356.6 (305.7–413.1)	0.15 (0.11–0.18)
5-9 years	1757.8 (1483.6–2061.8)	301.2 (254.2–353.3)	2474.4 (2120.0–2808.0)	360.2 (308.6–408.7)	0.66 (0.62–0.7)
10-14 years	1507.5 (1265.4–1799.3)	281.4 (236.2–335.9)	2397.0 (2024.3–2795.0)	359.5 (303.7–419.3)	0.88 (0.84–0.93)
<15	5392.6 (4646.8–6313.6)	309.7 (262.6-365.6)	7218.2 (6296.3–8113)	358.7 (413.5–306)	0.54 (0.5–0.58)

**SDI**					

High	563.6 (480.7–654.3)	303.2 (259.2–356.2)	521.1 (474.8–576.3)	301.6 (271–334.6)	0 (−0.06 to 0.07)
High-middle	821 (678.5–987.4)	300.2 (248.4–365.3)	692.8 (619.5–763.1)	299 (261–333.9)	0.11 (0.02–0.19)
Middle	1609.5 (1354.8–1944.4)	279 (231.4–342.1)	1966.9 (1733.6–2196.8)	346.7 (297.6–393.8)	0.83 (0.72–0.93)
Low-middle	1779.3 (1527.1–2058.3)	375.2 (312.9–445.2)	2365.2 (1977.9–2751.4)	407.6 (331.6–496.3)	0.3 (0.25–0.34)
Low	613.7 (516.2–726.7)	262.1 (214.8–315.9)	1666.3 (1464–1875.7)	361.9 (309.7–418.3)	1.16 (1.02–1.29)

**Region**					

High-income Asia Pacific	85.9 (69.6–106.2)	244.1 (197.3–303.1)	53.6 (48.9–58.7)	238.7 (217.3–262)	−0.03 (−0.06 to 0)
High-income North America	210.3 (179.8–244)	341 (283.2–402.2)	216.4 (190.9–244.1)	328.9 (282.5–373.7)	−0.15 (−0.25 to −0.05)
Western Europe	200.7 (168.1–242.7)	282.7 (236–342.8)	187.9 (170.3–207.8)	275.7 (248.8–306.8)	−0.03 (−0.07 to 0)
Australasia	15.7 (13.5–18)	341.3 (288–397.1)	16 (14.6–17.7)	279.1 (251.4–310.4)	−0.72 (−0.75 to −0.69)
Andean Latin America	47 (39.3–56.9)	315.9 (263.8–383.5)	63.8 (58.9–69.3)	352.5 (324.3–383.5)	0.54 (0.45–0.62)
Tropical Latin America	121.8 (97.7–152)	228.4 (182.6–286.1)	197 (178–216.2)	392.6 (351.5–434.9)	2.08 (1.96–2.2)
Central Latin America	217.7 (168.9–293.8)	337.8 (260.6–456)	255.3 (233.7–278.2)	402.5 (368–439.8)	0.59 (0.53–0.65)
Southern Latin America	49.8 (43.4–57.8)	333.4 (280.3–394.9)	54.2 (47.9–59.9)	371.5 (315.1–415.7)	0.4 (0.36–0.45)
Caribbean	54.1 (48.3–60.6)	474.4 (413.6–542.6)	52.3 (46.5–57.4)	453.7 (393.3–509.6)	−0.09 (−0.13 to −0.05)
Central Europe	114.9 (94.4–140)	386.8 (314–477.7)	65.9 (58.6–73.4)	369.5 (319.7–421.9)	−0.13 (−0.18 to −0.09)
Eastern Europe	162.8 (130.3–200.8)	315.2 (242.2–401.9)	105.5 (82.1–124.3)	292.9 (218.4–359.6)	−0.29 (−0.36 to −0.22)
Central Asia	74.8 (60.5–92)	298.5 (240.8–367.9)	100.1 (91–109.5)	361.8 (326.4–400.1)	0.78 (0.73–0.83)
North Africa and Middle East	528.9 (447.3–623.3)	375.3 (316.7–443.6)	732.8 (664.9–803.7)	399.9 (355.5–442.5)	0.32 (0.28–0.36)
South Asia	1994.2 (1673.7–2358.4)	458.5 (367.7–561.9)	2152.4 (1662–2728.9)	423.5 (315.9–570.6)	−0.26 (−0.37 to −0.14)
Southeast Asia	477.8 (404.1–572.1)	280 (230.2–340.2)	612.5 (542.7–674.7)	354.3 (304–398.3)	0.8 (0.73–0.87)
East Asia	638.1 (487.6–862.5)	193.5 (147.5–261.3)	643.2 (577.3–712.7)	240.3 (212.1–267.5)	0.98 (0.82–1.13)
Oceania	8.9 (7.7–10.1)	331.1 (279.6–389.8)	17 (15–18.8)	333.7 (284.2–377)	0.02 (−0.03 to 0.08)
Western Sub-Saharan Africa	124.9 (97.7–159)	135.5 (104.2–173.6)	647.2 (565.1–730.5)	299.4 (259.1–341.8)	2.81 (2.45–3.18)
Eastern Sub-Saharan Africa	160.9 (124.2–205.7)	172.4 (132.2–222.1)	745.3 (652.9–836.9)	418 (355.3–485)	3.38 (3.18–3.58)
Central Sub-Saharan Africa	39.3 (30.7–50.5)	147.3 (114.6–190.1)	187.9 (163.9–214)	319.6 (276.8–366.6)	2.99 (2.52–3.46)
Southern Sub-Saharan Africa	64.3 (48.6–83.7)	309.8 (231.8–413.5)	111.9 (94.8–128.3)	464.2 (377.2–549.7)	1.47 (1.25–1.68)

EAPC = estimate annual percentage changes; ASPR = age-standardized prevalence rate; CI = confidence interval; SDI = sociodemographic index; UI = uncertainty interval.

In 2021, there were 2.3 million (95% UI: 1.5 to 3.2) years lived with disability (YLDs), and the ASR of YLDs was 115.58 (95% UI: 75.65 to 161.52) (Table S1). From 1990 to 2021, the ASR of YLDs slightly increased, with an EAPC of 0.1 (95% CI: 0.06 to 0.14) (Table S1 and Figure 1). The ASR of YLDs also increased rapidly from 1995 to 2010, rising from 112.8 in 1995 to 118.8 in 2010.

Additionally, the ASPR and YLDs were observed to reach their lowest point in recent years in 2020. However, as this study only incorporated data from two years (2020 and 2021) during the COVID-19 pandemic, it remains challenging to clearly delineate the temporal trend in disease burden over this period.

### Sex disparity in the global burden

In 2021, there were 4.0 million (95% UI: 3.5 to 4.5) cases of epilepsy attributable to neonatal disorders in boys and 3.2 million (95% UI: 2.8 to 3.5) cases in girls (Table 1).

**Figure 2:**
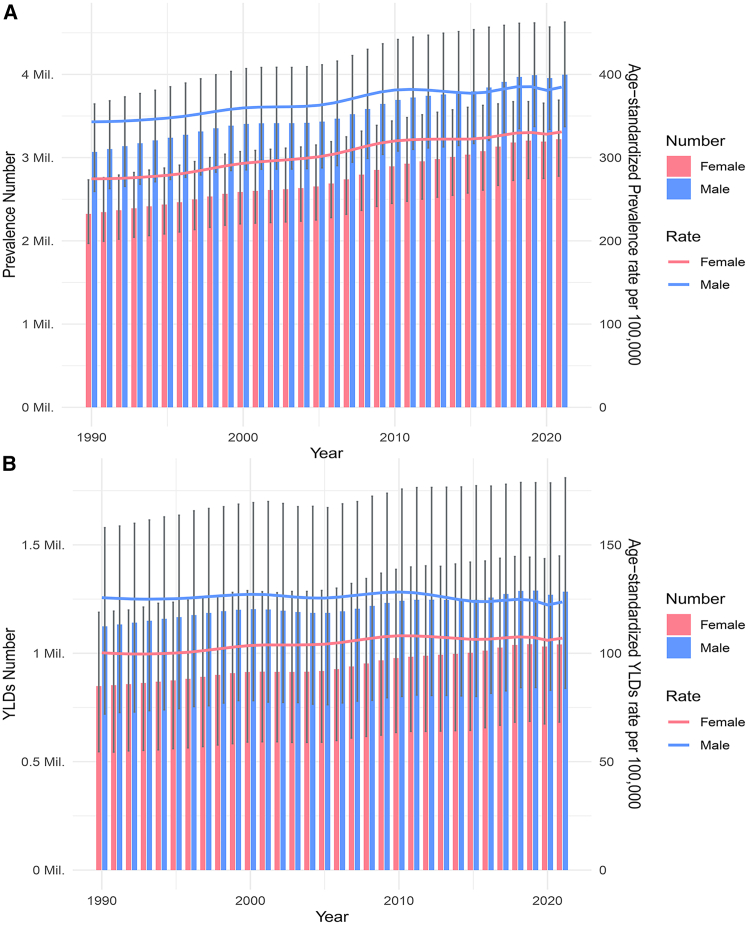
Global temporal trends for numbers and age-standardized rates from 1990 to 2021 for different sexes (A) Prevalence. (B) YLDs. Abbreviations: YLDs = years lived with disability. The estimated value is represented by the median of the data distribution, and the 95% uncertainty interval is represented by the 2.5th percentile and the 97.5th percentile. Error bars show the upper and lower limits of the uncertainty interval.

In 2021, there were 1.3 million (95% UI: 0.8 to 1.8) YLDs in boys and 1.0 million (95% UI: 0.7 to 1.4) in girls (Table S1). The ASR of YLDs in boys was 123.66 (95% UI: 80.79 to 174.17) and that in girls was 106.97 (95% UI: 70.19 to 148.89) (Table S1). The number and ASRs of YLDs were both greater in boys than in girls (Figures 2B and S1). From 1990 to 2021, the ASR of YLDs in boys remained stable, with an EAPC of −0.03 (95% CI: −0.07 to 0.01). In girls, the ASR of YLDs increased, with an EAPC of 0.28 (95% CI: 0.23 to 0.32) (Figure S1 and Table S1).

### Global burden stratified by age groups

The children were categorized into three age groups: 0 to 4 years, 5 to 9 years, and 10 to 14 years. In 2021, the number of cases and ASPRs of epilepsy attributable to neonatal disorders in children were similar across the three age groups (Table 1 and Figure S2B). From 1990 to 2021, the ASPR increased in all age groups. However, the increase was lowest in children aged 0 to 4 years and highest in children aged 10 to 14 years (Table 1 and Figure S2B).

In 2021, the number and ASR of YLDs across the three age groups were also approximately equal (Table S1 and Figure S2D). From 1990 to 2021, the ASR of YLDs among children aged 0 to 4 years decreased, with an EAPC of −0.31 (95% CI: −0.36 to −0.27). However, the ASR of YLDs increased in other age groups, especially among children aged 10 to 14 years (EAPC = 0.48, 95% CI: 0.44 to 0.53) (Table S1 and Figure S2D).

### Burden stratified by the SDI

**Figure 3:**
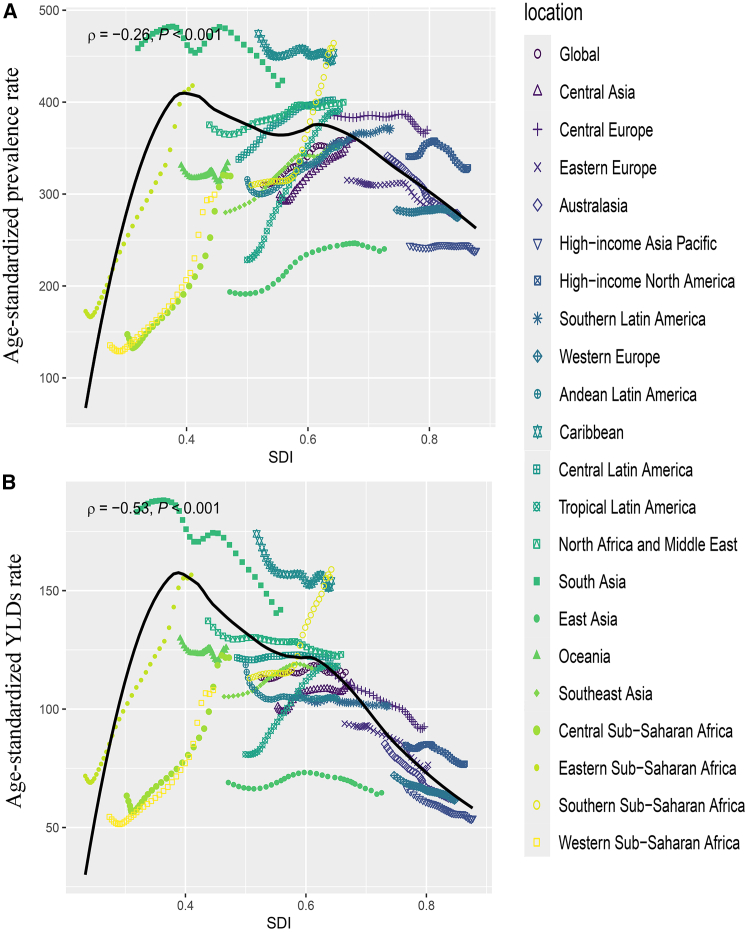
Trends in the age-standardized rates of 21 regions by SDI from 1990 to 2021 (A) Age-standardized prevalence rate (ASPR). (B) Age-standardized YLD rate. Abbreviations: YLDs = years lived with disability. The estimated value is represented by the median of the data distribution.

The ASPR of epilepsy attributable to neonatal disease in children varied significantly according to the SDI. The ASPR was greater in regions with SDIs ranging from 0.4 to 0.6. In regions with an SDI <0.4, the ASPR increased markedly with increasing SDI, particularly in African regions (Central Sub-Saharan Africa, Eastern Sub-Saharan Africa, Southern Sub-Saharan Africa, and Western Sub-Saharan Africa) and Tropical Latin America (Figures 3A and S3A).

The ASPR of the disease also varied across different SDI subgroups. In 2021, the low-middle-SDI regions had the highest number of cases and ASPRs of epilepsy attributable to neonatal disease, followed by the low-SDI regions. In contrast, regions with high or high-middle SDIs had lower case numbers and ASPRs (Table 1 and Figure S4). From 1990 to 2021, low-SDI regions presented the most significant increase in the ASPR (EAPC: 1.16; 95% CI: 1.02 to 1.29), whereas high-SDI and high-middle-SDI regions presented low ASPRs that remained stable (Table 1 and Figure S4).

The variation in the YLDs was similar to that in the prevalence. Regions with low-middle SDIs had high case numbers and ASRs of YLDs in 2021, and the ASR of YLDs increased sharply in low-SDI regions from 1990 to 2021.

### Burden stratified by GBD regions and nations

**Figure 4:**
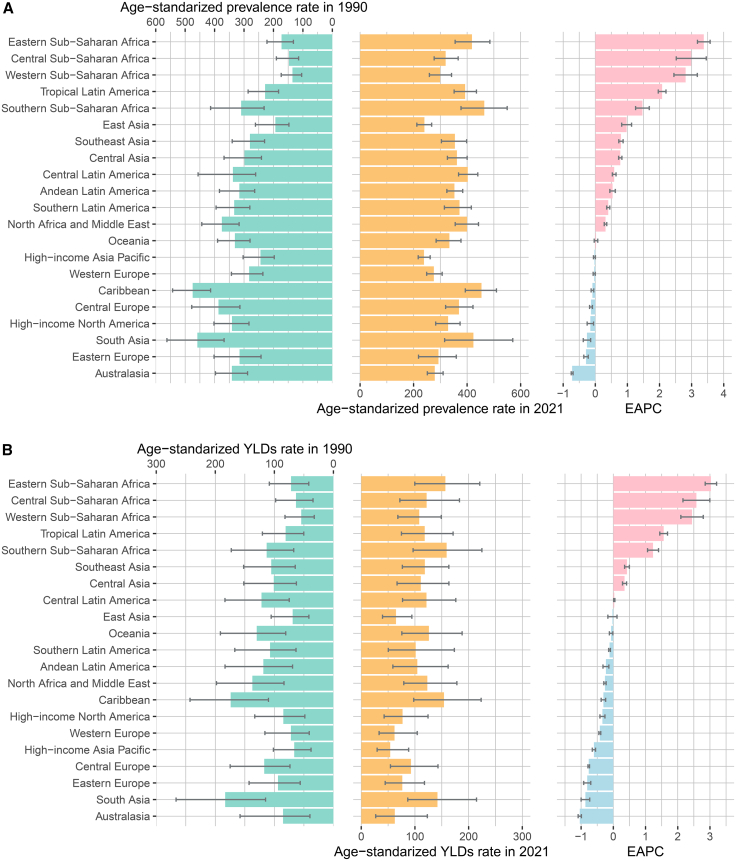
Regional age-standardized rates in 1990 and 2021 and their estimated annual percentage changes from 1990 to 2021 (A) Age-standardized prevalence rates in 1990 and 2021 and their estimated annual percentage changes. (B) Age-standardized YLD rates in 1990 and 2021 and their estimated annual percentage changes. Abbreviations: YLDs = years lived with disability. The estimated value is represented by the median of the data distribution, and the 95% uncertainty interval is represented by the 2.5th percentile and the 97.5th percentile. Error bars show the upper and lower limits of the uncertainty interval.

In 2021, the region with the highest prevalence of and YLDs from epilepsy attributable to neonatal disorders in children was South Asia and Australasia had the lowest prevalence and YLDs (Figure S5, Table 1, and S2). The region with the highest ASPR and YLDs in 2021 was Southern Sub-Saharan Africa, while the lowest rates were observed in the high-income Asia Pacific region (Figure 4, Tables 1, and S2). From 1990 to 2021, among the 21 regions, the greatest increases in the ASPR and YLDs were observed in Eastern Sub-Saharan Africa, whereas Australasia experienced the most rapid decreases (Figure 4 and Table 1).

**Figure 5:**
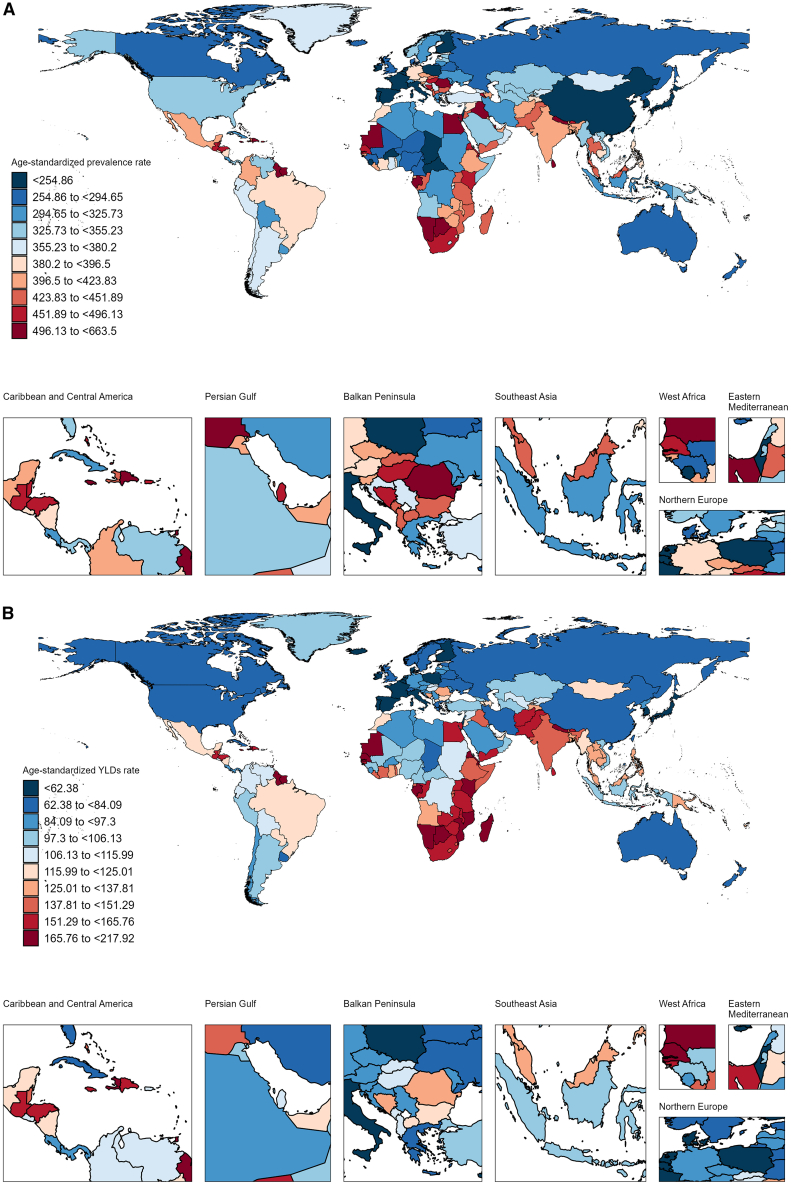
Summary map of national age-standardized rates in 2021 (A) Age-standardized prevalence rate (ASPR). (B) Age-standardized YLD rate. Note: The original data were obtained from the GBD database. Regional divisions follow the GBD study conventions. Abbreviations: YLDs = years lived with disability. The estimated value is represented by the median of the data distribution.

In 2021, among the 204 countries, India had the highest prevalence of and YLDs from epilepsy attributable to neonatal diseases in children (Figure S6, Tables S3 and S4). Trinidad and Tobago and Botswana had the highest ASPR and YLDs, whereas Belgium had the lowest ASPR and YLDs (Figure 5, Tables S3 and S4).

From 1990 to 2021, Ethiopia had the largest increase in the ASPR and YLDs, followed by Nigeria and Liberia, while Nepal and the Maldives had the greatest decreases (Figure S7, Tables S3 and S4).

### Burden attributable to four kinds of neonatal disorders

**Figure 6:**
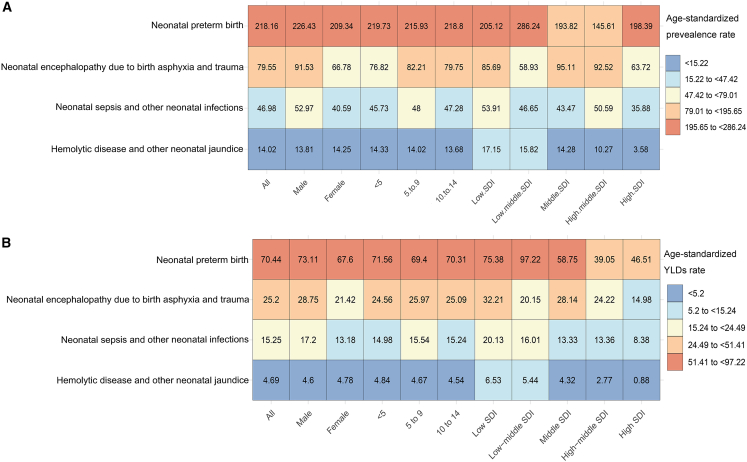
Heatmap of age-standardized rates of childhood epilepsy attributable to four types of neonatal disorders in 2021 (A) Age-standardized prevalence rate (ASPR). (B) Age-standardized YLD rate. Abbreviations: YLDs = years lived with disability. The estimated value is represented by the median of the data distribution.

In 2021, epilepsy due to preterm birth accounted for 61% of all epilepsy cases attributable to neonatal disorders in children, with a burden of 4.4 million (95% UI: 3.7 to 5.1) and an ASPR of 218.16 (95% UI: 179.2 to 260.86) (Tables S5 and S7). Additionally, encephalopathy and neonatal infections were significant causes of epilepsy, accounting for 22% and 13% of the cases, respectively. Hemolytic disease and other neonatal jaundice were the least prevalent, accounting for 4% of the cases (Table S7). After stratification by sex, age, and SDI, preterm birth remained the most common cause of childhood epilepsy, followed by encephalopathy and neonatal infections (Figure 6).

From 1990 to 2021, the severity of the four subtypes of neonatal disorders remained stable (Figure S8). However, the ASPR of neonatal encephalopathy and neonatal infections increased, with EAPCs of 1.92 (95% CI: 1.64 to 2.2) and 1.11 (95% CI: 0.9 to 1.33), especially in regions with low or low-middle SDIs (Table S6). Within each population group, the order of neonatal diseases that cause epilepsy was the same (Figure S9). The distribution of different neonatal disorders in YLDs was similar to that of the ASPR.

## Discussion

In the present study, we examined the global, regional, and national burdens of epilepsy attributable to neonatal disorders in children and the temporal trends from 1990 to 2021 using the GBD 2021 dataset. While the burden of childhood epilepsy has previously been demonstrated,^10^ epilepsy specifically attributable to neonatal disorders and its spatiotemporal distribution pattern have not been depicted. Our study provides a comprehensive global assessment of childhood epilepsy attributable to neonatal disorders and depicts the disparity of the disease burden in populations stratified by sex, age group, SDI, region, and nation.

The global burden of the disease remains a serious concern. In 2021, there were 7.2 million cases of childhood epilepsy attributable to neonatal disorders, with an ASPR of 358.7. As reported in a previous study, the total prevalence of childhood epilepsy is 13.6 million.^1^ Neonatal disorders are responsible for 53% of all epilepsy cases in children, which is similar to the proportion of YLDs attributed to neonatal disorders. Our result revealed that the presence of neonatal disorders is the most important risk factor for childhood epilepsy and emphasized the necessity of controlling neonatal disorders to protect against the development of epilepsy in children. The EAPC and joinpoint models demonstrated that from 1990 to 2021, the ASPR increased, whereas the ASR of YLDs remained stable. Considering the improvements of diagnostic techniques and the survival rate of high-risk newborns, it is reasonable to identify an increasing trend of epilepsy attributable to neonatal disorders.^11^ As mentioned in the Methods, YLDs were calculated as the prevalence × disability weight. With improved global access to treatment and therapeutic outcomes, the prognosis for epilepsy has significantly improved, leading to a decrease in its disability weight. However, the decrease in disability weight and the increase in the number of patients have offset one another, resulting in a stable value for YLDs over the past three decades.

From 1990 to 2021, the prevalence rate of all-cause epilepsy increased, with an EAPC of 0.17. In the same period, the EAPC of epilepsy attributable to neonatal disorders was 0.54, which is approximately 3 times higher than that of all-cause childhood epilepsy. Notably, the YLDs for childhood epilepsy decreased from 1990 to 2021, with an EAPC of −0.27, while the YLDs for childhood epilepsy attributable to neonatal disorders remained stable. Therefore, neonatal disorders were found to be the primary impetus of the increase in childhood epilepsy.

Notably, the ASPR and YLDs both increased from 1995 to 2010, which indicates that the burden of epilepsy attributable-income and high-income countries. Advances in medical technology have enabled the survival of high-risk newborns who might not have survived in the past, but these “survivors” are also at high risk for future neurological sequelae, including epilepsy. The expansion of the survival base directly leads to an increase in subsequent epilepsy case statistics.^3^

We also found that the burden of disease reached its lowest point upon the onset of the COVID-19 pandemic in 2020. This can be attributed to forced delay in early screening and diagnosis for neurological complications of neonatal disorders due to the COVID-19 pandemic.^12^

The ASPR and YLDs for childhood epilepsy attributable to neonatal disorders were elevated in boys. Similarly, previous epidemiological studies utilizing data from the GBD study reported that epilepsy is more prevalent among male patients.^13^^,^^14^ The cause of the sex difference remains unclear and may be attributed to important biological, environmental, and behavioral factors. For example, in regions such as India and South Africa, poor social attitudes contribute to lower health concerns and follow-up rates among girls, which may underestimate the burden of the disease.^15^^,^^16^ Additionally, differences in steroid hormone levels between males and females may increase boys' susceptibility to seizures attributable to neonatal illness, thereby contributing to the observed sex differences.^17^^,^^18^

Numerous prior studies have indicated that children aged 0 to 4 years are particularly susceptible to epilepsy.^19^^,^^20^ The prevalence and YLDs of epilepsy have increased in this age group.^1^^,^^21^ Our analysis showed that while the heaviest disease burden was historically among children aged 0–4 years, it has now become comparable across all age groups. In contrast to previous studies, the present analysis incorporates the most recent global epidemiological data and characterizes the temporal changes in disease burden across different age groups. Notably, in children aged 5–15 years, the ASPR and YLDs for epilepsy attributable to neonatal disorders have increased in the past 30 years, surpassing those reported in children aged 0 to 4 years. This trend indicates that long-term neurological complications in later childhood resulting from neonatal disorders have emerged as critical issues that require urgent attention. Previous studies have also reported that neonatal disorders and conditions such as preterm birth, perinatal stroke, and HIE are significant risk factors for epilepsy in later childhood and adulthood.^22^^,^^23^^,^^24^ Our study underscores the critical importance of preventative measures and treatment strategies to address long-term complications associated with neonatal disorders.

The burden of childhood epilepsy attributable to neonatal disorders exhibited significant regional variability. The regional variability may be partly accounted for by the difference in the SDI. Our study revealed that the burden of the disease is greater in low-SDI regions than in high-SDI regions. Furthermore, low-SDI regions have experienced a significant increase in the burden of epilepsy, whereas the burden in high-SDI and high-middle-SDI regions has remained relatively low over the past three decades. Our findings suggest that a lack of medical resources may aggravate the burden of epilepsy in children with neonatal disease. In high-SDI regions, effective neonatal care and medical interventions can substantially reduce the risk of neonatal disorders and associated complications, including epilepsy.^25^ Consistent with our results, previous studies revealed that the incidence of epilepsy is greater in low-income countries than in high-income countries.^26^^,^^27^ Medical infrastructure in lower-SDI regions should be strengthened to mitigate the burden of neonatal disorder-related epilepsy. Our study is one of the first to elucidate the association between the burden of childhood epilepsy attributable to neonatal disorders and SDI levels. In addition, we found that the burden of childhood epilepsy varies across regions with similar SDIs, which may suggest that genetic variations also contribute to regional differences in the disease.^21^

From 1990 to 2021, preterm birth was identified as the predominant cause of childhood epilepsy in population groups stratified by sex, age, and SDI. A Danish population-based cohort study of 1.4 million singletons confirmed an association between preterm birth and an elevated risk of epilepsy in early life, which aligns with our findings.^28^ Previous studies have indicated that preterm birth and associated etiological factors, such as intrauterine infection, can precipitate HIE, thereby increasing the risk of epilepsy.^24^ Preterm birth can also result in diffuse white and gray matter abnormalities, which are associated with an increased risk of epilepsy.^29^

In conclusion, our study provides a thorough analysis of the temporal trends of epilepsy attributable to neonatal disorders in children and its distribution across various demographic subgroups. Globally, the disease burden increased from 1990 to 2021. In 2021, the burden was disproportionately greater in males and regions with low or low-middle SDIs, with the burden being relatively balanced across age groups. Furthermore, preterm birth was identified as the most prevalent pathogenic factor for epilepsy among the four types of neonatal disorders.

We anticipate that this study will provide policymakers with the necessary detailed information to allocate resources, implement effective measures, and foster interregional collaboration aimed at enhancing medical care for children with neonatal disorders in low-income countries. Moreover, our study underscores the importance of future research into advanced therapeutic options and preventive strategies for childhood epilepsy.

### Limitations of the study

Despite our efforts to perform a comprehensive analysis of epilepsy attributable to neonatal disorders in children, this study has certain limitations. First, the burden of pediatric epilepsy is likely underestimated in underdeveloped regions and socioeconomically disadvantaged populations, where epilepsy and neonatal disorder surveillance systems often have limited coverage. Second, GBD 2021 included only two years of data obtained during the COVID-19 pandemic, i.e., 2020 and 2021. We recognize that clearly identifying the complex, nonlinear, and long-term effects of the COVID-19 pandemic on epilepsy attributable to neonatal disorders is difficult. In the GBD 2023 dataset,^30^ the entire course of the COVID-19 pandemic was covered. This information is helpful for obtaining a full understanding of the impact of the COVID-19 pandemic on epilepsy attributable to neonatal disorders. Third, the GBD 2023 estimates have been released.^30^ Epidemiological features observed in the present data may change based on the new GBD 2023 dataset. Moreover, although numerous epidemiological studies have been conducted globally or at a national level, research focusing on epilepsy attributable to neonatal disorders remains scarce. Therefore, cross-validation of our findings with existing literature is challenging. In the future, we hope that multicenter prospective cohort studies can be conducted to elucidate the causality between various types of neonatal disorders and childhood epilepsy.

## Resource availability

### Lead contact

Further information and requests for resources should be directed to and will be fulfilled by the lead contact, Xin Tian (xintian@cqmu.edu.cn).

### Materials availability

This study did not generate new unique reagents.

### Data and code availability

Data: The data used in this study are available at Open Science Framework: https://doi.org/10.17605/OSF.IO/SQUK5, DOI: 10.17605/OSF.IO/SQUK5. The ULR and DOI for the repository are also listed in the key resources table.

Code: All original codes used in this study are available at Open Science Framework: https://doi.org/10.17605/OSF.IO/SQUK5, DOI:10.17605/OSF.IO/SQUK5. The ULR and DOI for the repository are also listed in the key resources table.

Any additional information required to reanalyze the data reported in this paper is available from the lead contact upon request.

## Acknowledgments

We thank the Global Burden of Disease Study 2021 collaborators for providing the most comprehensive analysis of different diseases on a global scale.

## Author contributions

S.Y.Z. and Y.H.L., conceptualization, formal analysis, investigation, writing – original draft, and writing – review and editing. H.Y., investigation and writing – review and editing. K.Y.J., investigation and writing – review and editing. N.N.Z., investigation and writing – review and editing. P.K., conceptualization, investigation, writing – review and editing, supervision, and funding acquisition. Y.Y., conceptualization, investigation, writing – review and editing, supervision, and funding acquisition. X.T., conceptualization, investigation, writing – review and editing, supervision, and funding acquisition.

The corresponding authors are responsible for the authenticity of the data. All authors contributed substantively to the work, read and approved the final manuscript, and accept full accountability for all aspects of the study.

## Declaration of interests

The authors declare no competing interests.

## STAR★Methods

### Key resources table

**Table:** 

REAGENT or RESOURCE	SOURCE	IDENTIFIER
**Software and algorithms**		

R 4.4.1 (64-bit)	The R Foundation for Statistical Computing	https://www.r-project.org/
R Studio 2024.04.1	Posit Software	https://posit.co/downloads/

**Other**		

GBD 2021	IHME	http://ghdx.healthdata.org/gbd-results-tool
Data and code	Open Science Framework	DOI: 10.17605/OSF.IO/SQUK5 URL: https://doi.org/10.17605/OSF.IO/SQUK5

### Experimental model and study participant details

All data used in this study were obtained from the Global Burden of Disease (GBD) database.

### Method details

#### Data sources

All epidemiological data concerning epilepsy attributable to neonatal disorders in children included in this study were sourced from the GBD 2021 database, which is publicly accessible via the IHME website: https://gbd2021.healthdata.org/gbd-results/. The basic information and data source of the GBD 2021 is described in the Supplementary Methods (1. Overview, 2. Data sources). Epilepsy attributable to neonatal disorders is coded as 8A60.0 in the ICD-11 (epilepsy caused by brain damage during the prenatal or perinatal period). The GBD 2021 considers four types of neonatal disorders and conditions that are associated with epilepsy: neonatal preterm birth, neonatal encephalopathy due to birth asphyxia and trauma, neonatal sepsis and other neonatal infections, and hemolytic disease and other neonatal jaundice. We collected global epidemiological data on children with epilepsy attributable to neonatal disorders, including the prevalence, number of YLDs, and demographic data (age, sex, location), spanning 1990 to 2021 in this study. Children were identified as individuals under 15 years old.^31^ The detailed procedure for extracting and downloading the data can be found in the Supplementary Methods (4. The procedure for extracting data on epilepsy attributable to neonatal disorders).

#### Estimates of the global burden

The GBD study employs a sophisticated and rigorous methodology to assess the burden of epilepsy attributable to neonatal disorders. The prevalence and YLDs, along with their quantified forms, number of cases and age-standardized rates (ASRs), were used to characterize the disease burden associated with epilepsy attributable to neonatal disorders. The prevalence and YLDs of disease are derived from the secondary analysis of an epidemiological report and a statistical model. We summarized this estimation method in the Supplementary Methods (3. Estimation of the epilepsy burden attributable to neonatal disorders). The YLDs, calculated as the Prevalence × Disability Weight, denotes the years of healthy life lost due to disability from illness or injury.^30^ Rates are expressed as the number of cases per 100,000 population. These data are presented as point estimates with 95% uncertainty intervals (UIs), including baseline, lower, and upper bounds derived from the 2.5th and 97.5th percentiles of 1000 resampled input data points for each quantity of interest.

Considering the regional differences around the world, we also describe the global disease burden at the regional and national levels. According to the GBD study, the official GBD methodology classifies the world into 7 super regions, 21 regions, and 204 countries and territories.^30^ In present study, we used GBD regions as the primary classification standard, which is also widely used in other GBD research.^32^^,^^33^^,^^34^ This strategy ensures consistency with the standard GBD classification framework, enhancing the precision and comparability of our spatial analysis.

#### Sociodemographic index (SDI)

The SDI is a comprehensive metric for assessing economic and developmental levels, which are closely associated with health outcomes.^7^ It integrates lag-distributed income per capita, the total fertility rate of females aged under 25 years, and average years of schooling among individuals aged 15 years and older. The SDI, which ranges from 0 to 1, categorizes world regions into five quintiles of development: low, low-middle, middle, high-middle, and high-SDI regions.

#### Estimates of the temporal trends

To evaluate the temporal trends in the burden of epilepsy attributable to neonatal disorders, we constructed a joinpoint model and calculated the estimated annual percentage change (EAPC) for the age-standardized prevalence rate (ASPR) and age-standardized YLD rate globally from 1990 to 2021.

Joinpoint models employ segmented lines and turning points derived from piecewise linear regression to ascertain temporal trends.^35^^,^^36^ Joinpoints, or turning points, were identified where a statistically significant trend change occurred. A maximum of six joinpoints was permitted. Trends for each period were assessed using annual percent changes (APCs).^37^

The use of a linear regression model to compute the EAPCs is a standard quantitative approach for evaluating long-term trends in ASRs over a defined period.^38^ The EAPCs are reported with their corresponding 95% confidence intervals (CIs). An EAPC above 0 suggests an increasing trend in the ASR, whereas an EAPC below 0 indicates a decreasing trend. The EAPC was used to analyze the global temporal trends in the burden of epilepsy attributable to neonatal disorders in children from 1990 to 2021, stratifying the data by sex, age, SDI, GBD subcontinental region, and country.

#### Patient engagement and protocol approvals

This study utilized openly collected data and conducted in-depth analyses without utilizing any personally identifiable information. The patients did not participate in determining the research questions, designing and executing the experiments, or measuring results; hence, patient consent was not necessary.

### Quantification and statistical analysis

All analyses were performed using R software (version 4.4.1). The study included individuals aged under 15 years. All estimates (prevalence, YLDs) are presented with 95% uncertainty intervals (UIs) from the GBD 2021 model. The unit of analysis is the population of 204 countries and territories from 1990 to 2021. Initially, we analyzed the burden and temporal trends of epilepsy attributable to neonatal diseases in children stratified by sex, age, SDI, region, and country. Temporal trends in age-standardized rates (ASPR, ASYR) were quantified using the estimated annual percentage change (EAPC), calculated by fitting the linear model ln(ASPR) = α + β × year and computing EAPC = 100 × (e^β^ - 1). An EAPC with 95% confidence interval (CI) > 0 indicates an increasing trend; < 0 indicates a decreasing trend. Joinpoint regression identified inflection points. Then, the relationships between ASPR and Sociodemographic Index (SDI) in 2021 were assessed using Pearson correlation, reporting the coefficient (r) and p-value. p < 0.05 was considered significant. Finally, we assessed and compared the burden of childhood epilepsy attributable to four important subtypes of neonatal disorders. R 4.4.1 (64-bit) software and associated packages were used for data collation, processing, result output, and diagram plotting. An analytic workflow is available in Supplementary Methods (5. Analytic workflow of our study).

## Supplemental information

Document S1. Figures S1–S9, Tables S1, S2, and S5–S7, and Methods S1

Table S3. Age-standardized prevalence rate and prevalence number of epilepsy attributable to neonatal disorders in children under 15 years and their EAPCs from 1990 to 2021 in 204 countries and territories

Table S4. Age-standardized YLDs rate and YLDs number of epilepsy attributable to neonatal disorders in children under 15 years and their EAPCs from 1990 to 2021 in 204 countries and territories
