# Supplementary material for: Global burden of epilepsy attributable to neonatal disorders in children from 1990 to 2021
Source: iScience. 2026 Mar 10;29(4):115296. doi: 10.1016/j.isci.2026.115296 (PMC13049413; doi:10.1016/j.isci.2026.115296)
Supplement: Document S1. Figures S1–S9, Tables S1, S2, and S5–S7, and Methods S1 [file mmc1.pdf]

iScience, Volume *29*

Supplemental information

Global burden of epilepsy attributable to neonatal disorders in children from 1990 to 2021

Shanying Zhong, Yihe Lian, He Yi, Kaiyun Jia, Ningning Zhang, Patrick Kwan, Yong Yang, and Xin Tian

Supplementary Figures

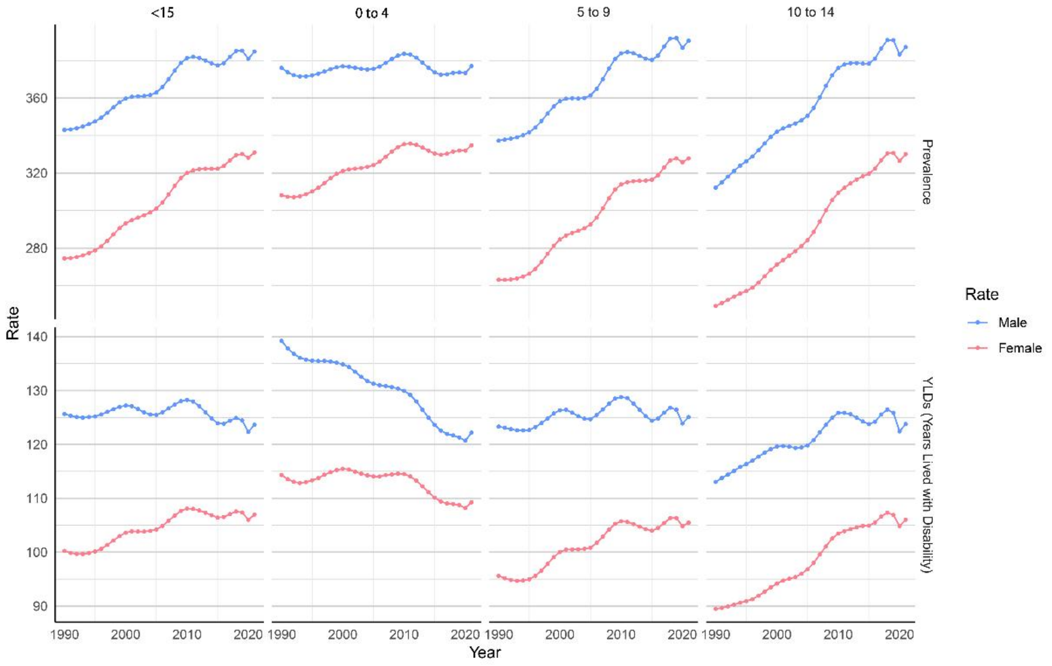

Supplementary Figure 1: Temporal trend of epilepsy burden attributable to neonatal disorders in children under 15 years among different sex, stratified by different age from 1990 to 2021. The estimated value is represented by the median of the data distribution. Data are presented as mean ± SEM.

Abbreviations:

YLDs: Year lived with disability

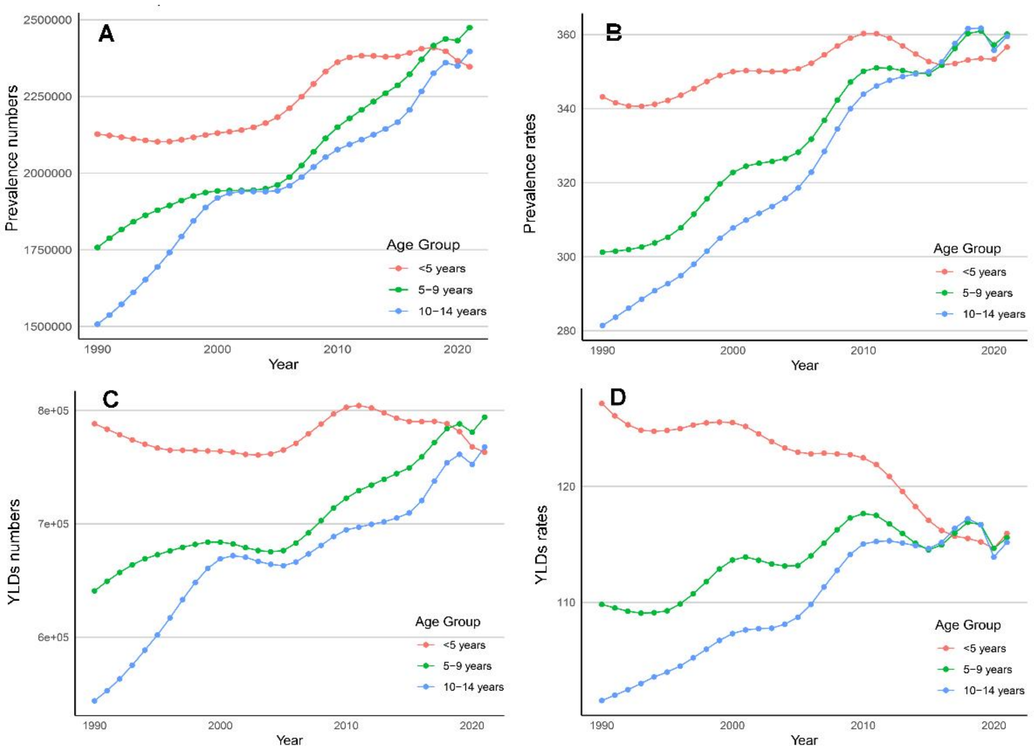

Supplementary Figure 2: Temporal trend of epilepsy burden attributable to neonatal disorders in children under 15 years among 3 age groups from 1990 to 2021. The estimated value is represented by the median of the data distribution. Data are presented as mean ± SEM.

(A): Prevalence number

(B): Prevalence rate

(C): YLDs number

(D): YLDs rate

Abbreviations:

YLDs: Year lived with disability

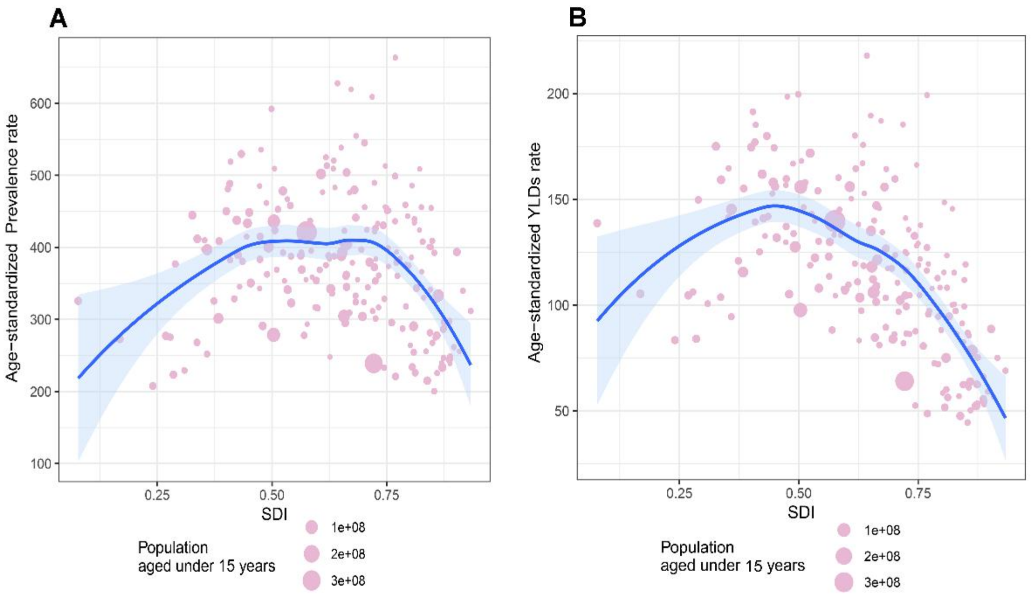

Supplementary Figure 3: The association between socio-demographic index (SDI) and the burden of epilepsy attributable to neonatal disorders in children under 15 years in 2021. The estimated value is represented by the median of the data distribution. Data are presented as mean ± SEM.

(A): Age-standardized prevalence rate

(B): Age-standardized YLDs rate

Abbreviations:

YLDs: Year lived with disability

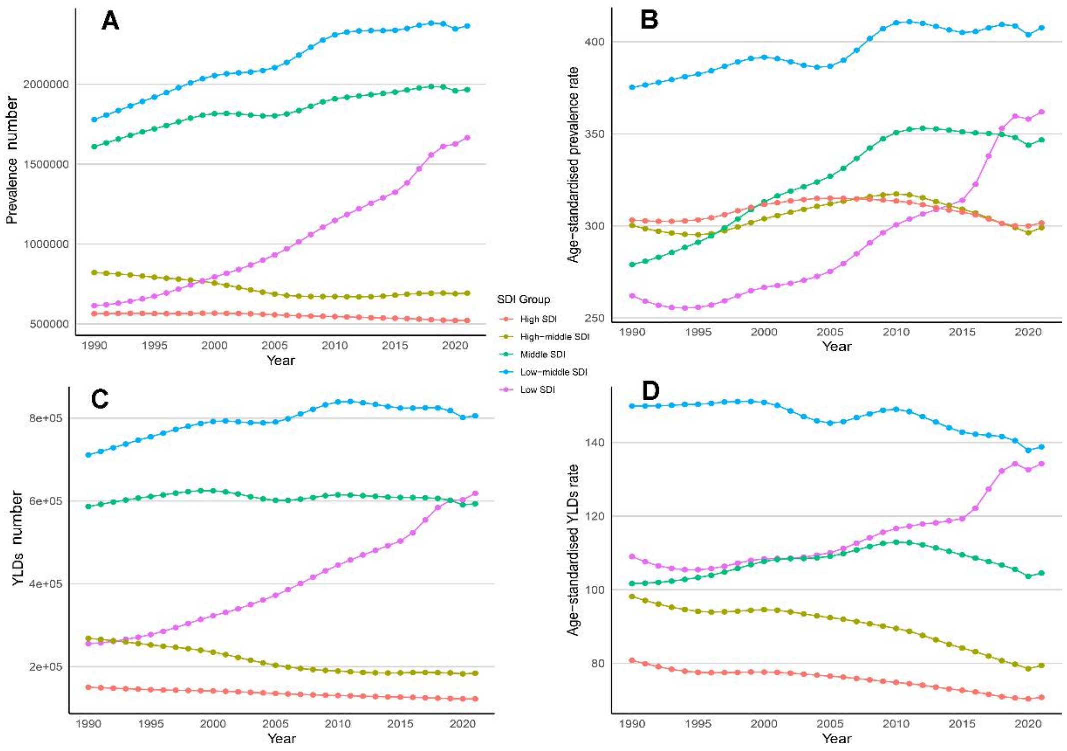

Supplementary Figure 4: Temporal trend of epilepsy burden attributable to neonatal disorders in children under 15 years among five SDI quintiles from 1990 to 2021. The estimated value is represented by the median of the data distribution. Data are presented as mean ± SEM.

(A): Prevalence number

(B): Age-standardized prevalence rate

(C): YLDs number

(D): Age-standardized YLDs rate

Abbreviations:

YLDs: Year lived with disability

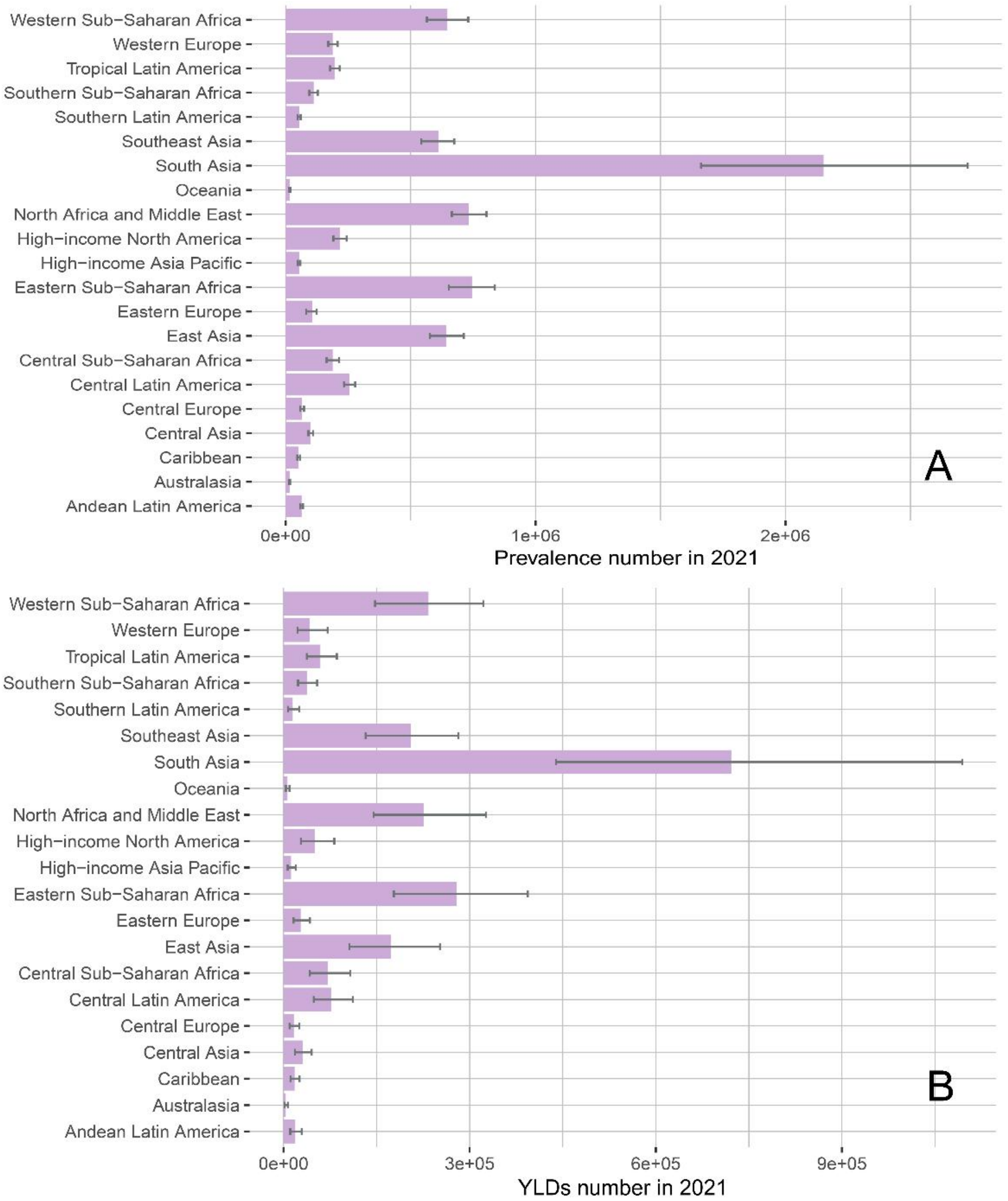

Supplementary Figure 5: The burden of epilepsy attributable to neonatal disorders in children under 15 years among 21 regions in 2021. The estimated value is represented by the median of the data distribution, and the 95% uncertainty interval is represented by the 2.5th percentile and the 97.5th percentile. Error bars show the upper and lower limits of the uncertainty interval. Data are presented as mean ± SEM.

(A): Prevalence number.

(B): YLDs number.

Abbreviations:

YLDs: Year lived with disability

.

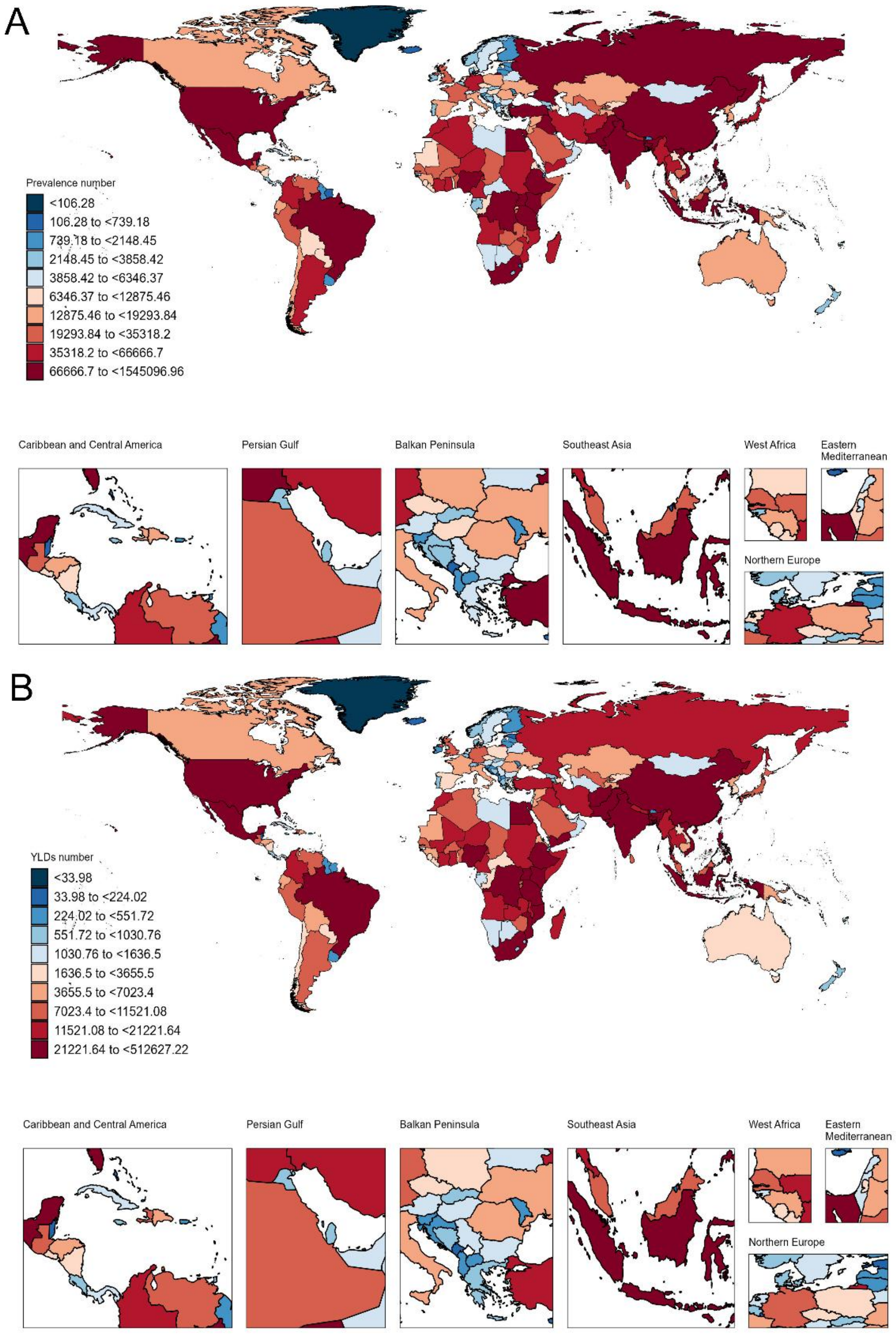

Supplementary Figure 6: The burden of epilepsy attributable to neonatal disorders in children under 15 years in 204 countries in 2021. The estimated value is represented by the median of the data distribution. Data are presented as mean ± SEM.

(A): Prevalence number.

(B): YLDs number. Abbreviations: YLDs: Year lived with disability

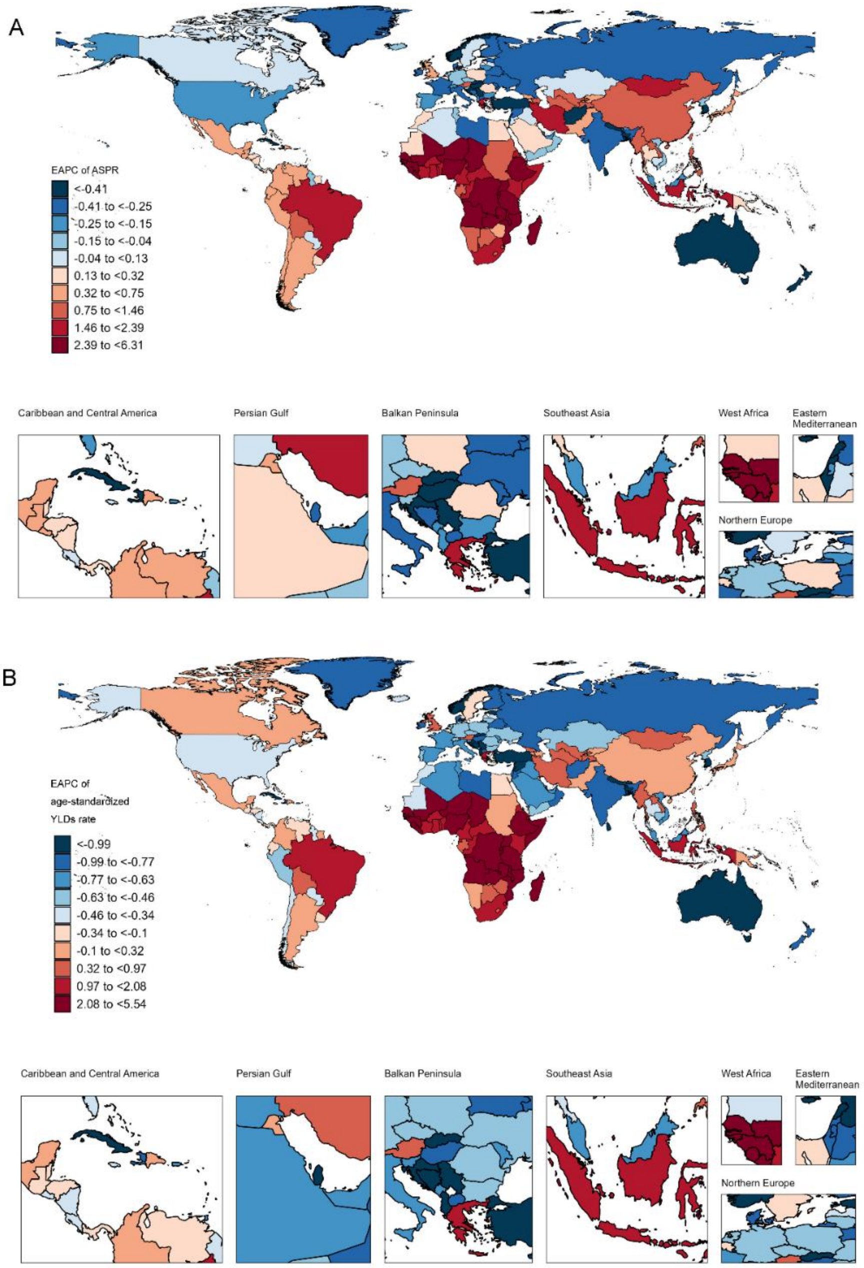

Supplementary Figure 7: The estimated annual percentage changes of the burden of epilepsy attributable to neonatal disorders in children under 15 years among 204 countries. The estimated value is represented by the median of the data distribution. Data are presented as mean ± SEM.

(A): Estimated annual percentage change of age-standardized prevalence rate

(B): Estimated annual percentage change of age-standardized YLDs rate

Abbreviations: EAPC: estimated annual percent change

ASPR: age-standardized prevalence rate

YLDs: Year lived with disability

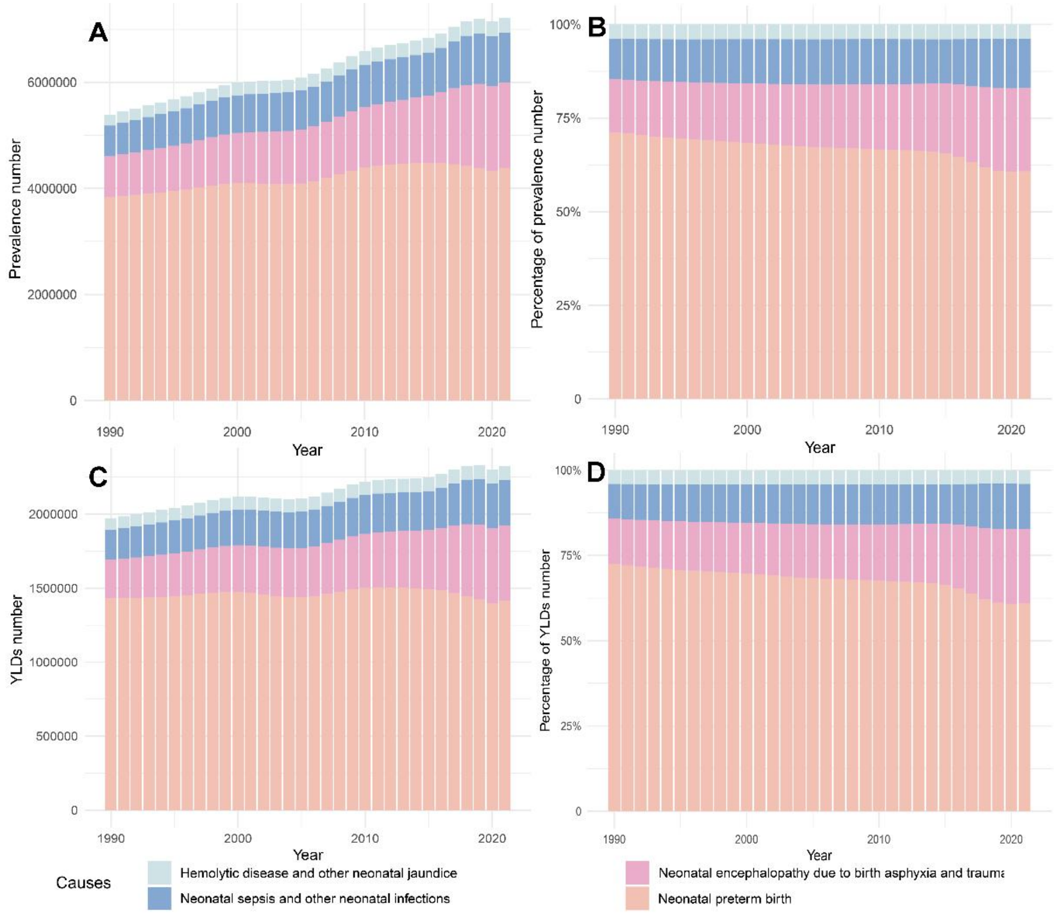

Supplementary Figure 8: Temporal trend of epilepsy burden attributable to four neonatal disorders in children under 15 years. The estimated value is represented by the median of the data distribution. Data are presented as mean ± SEM.

(A): Prevalence number

(B): Percentage of prevalence number

(C): YLDs number

(D): Percentage of YLDs number

Abbreviations:

YLDs: Year lived with disability

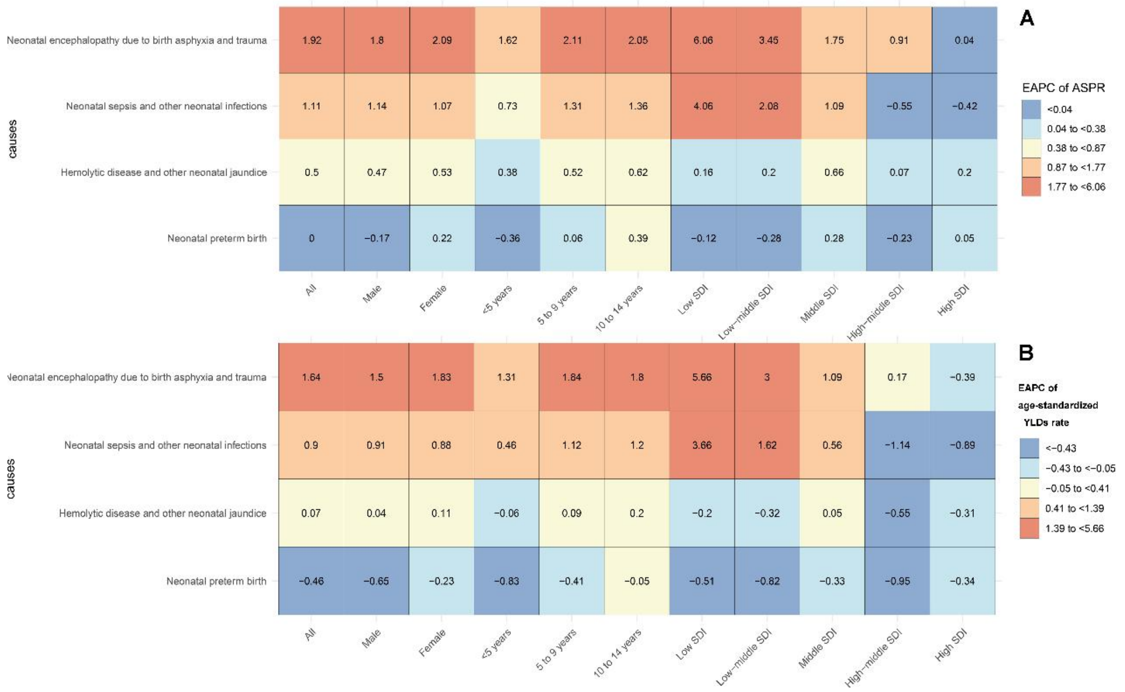

Supplementary Figure 9: The estimated annual percentage changes of the burden of epilepsy attributable to four important neonatal disorders in different subgroups of people under 15 years. The estimated value is represented by the median of the data distribution. Data are presented as mean ± SEM.

(A): Estimated annual percentage change of age-standardized prevalence rate

(B): Estimated annual percentage change of age-standardized YLDs rate

Abbreviations:

EAPC: estimated annual percent change

ASPR: age-standardized prevalence rate

YLDs: Year lived with disability

Methods S1: Detailed procedures for data extraction and burden estimation of epilepsy attributable to neonatal disorders, Related to STAR Methods

1. Overview

The Global Burden of Disease (GBD) represents a global descriptive epidemiology approach. It systematically quantifies the comparative health loss from diseases, injuries, and risk factors across age, sex, and geography at specific times. The Institute for Health Metrics and Evaluation (IHME) acts as the central coordinating body for the GBD and associated projects. GBD 2021 offers independent population estimates for 204 countries and territories, and globally, employing a standardized, replicable methodology, along with updates on fertility and migration. GBD 2021 includes significant data expansions, enhancements, and methodological advancements. The estimates for mortality and life expectancy now cover 983 locations with the highest level of detail, with additional causes incorporated into the lists of fatal and nonfatal conditions, totaling 371 diseases and injuries. (http://www.healthdata.org/gbd/about/protocol). GBD 2021 assessed key epidemiological metrics—including incidence, prevalence, mortality, YLDs, YLLs, and DALYs—across 23 age groups, for males, females, and both sexes combined, in 204 countries and territories, categorized into 21 regions and seven super-regions. The GBD 2021 location hierarchy encompasses all World Health Organization (WHO) member states. The GBD's disease and injury analytical framework produced estimates annually from 1990 to 2021. Diseases and injuries are categorized within a tiered cause hierarchy, ranging from three overarching causes at Level 1 to the most detailed at Level 4. Under the three Level 1 categories—communicable, maternal, neonatal, and nutritional diseases; noncommunicable diseases; and injuries—the framework includes 22 Level 2 categories, 174 Level 3 categories, and 301 Level 4 categories (with 131 Level 3 categories not further detailed at Level 4). Collectively, there are 365 nonfatal and 288 fatal causes.

2. Data sources

GBD 2021 integrates an extensive and expanding array of data sources, encompassing surveys, censuses, vital statistics, and additional health-related datasets. Data from these sources estimate morbidity, illness, injury, and attributable risk for 204 countries and territories from 1990 to 2021, while mortality estimates span from 1980 to 2021. The GBD estimation process involves identifying various pertinent data sources for each disease or injury, such as censuses, household surveys, civil registration and vital statistics, disease registries, health service utilization, air pollution monitoring, satellite imagery, disease notifications, and more. These data types are sourced through systematic reviews of scholarly articles, web searches of governmental and international organization sites, published reports, primary sources like the Demographic and Health Surveys, and dataset contributions from GBD collaborators. All study data were sourced from the Global Health Data Exchange (http://ghdx.healthdata.org/gbd-results-tool) comprising: (1) global age- and sex- specific prevalence, YLDs, and crude rates (per 100,000 persons) from 1990 to 2021; (2) regional age- and sex- specific prevalence, YLDs, and crude rates

from 1990 to 2021, categorized by SDI; (3) national age- and sex- specific prevalence, YLDs, and crude rates from 1990 to 2021; (4) GBD's 2017 world standard population.

3. Estimation of the epilepsy burden attributable to neonatal disorders.

The Global Burden of Disease (GBD) 2021 study conducted a comprehensive systematic review to ascertain the global burden of epilepsy impairments. Representative, population-based surveys reporting on prevalence, incidence, remission rate, excess mortality rate, relative risk of mortality, standardized mortality ratio, and comorbidity-adjusted mortality rate were included in the study. Surveys lacking a well-defined sample population were excluded (e.g., those focusing on clinic attendees or patient organization members with unspecified or non-representative geographical coverage). Data from various sources were standardized utilizing the Cause of Death Ensemble model, spatiotemporal Gaussian process regression, and DisMod-MR.

More details of data analysis in GBD 2021 can be found in study “Global incidence, prevalence, years lived with disability (YLDs), disability-adjusted life-years (DALYs), and healthy life expectancy (HALE) for 371 diseases and injuries in 204 countries and territories and 811 subnational locations, 1990-2021: a systematic analysis for the Global Burden of Disease Study 2021” and “Global, regional, and national burden of epilepsy, 1990–2016: a systematic analysis for the Global Burden of Disease Study 2016” and their appendix documents.

The burden of epilepsy attributable to neonatal disorders was elevated by systematic literature review and strict statistical models. We will summarize this estimation method as follows.

In the first step, prevalence and total YLD of epilepsy were calculated. GBD has its own independent model to estimate the total prevalence and total YLD of epilepsy globally, by country, and by age and sex group. This calculation does not take etiology into account. By integrating all available epilepsy survey data, hospitalization records, literature, etc., disease modeling tools (such as DisMod-MR) were used to estimate a highly accurate prevalence rate and YLD rate.

In the second step, the proportion of prevalence and YLD rate attributable to neonatal diseases were calculated. GBD researchers first confirmed through systematic literature reviews and meta-analyses that neonatal diseases are strong risk factors for the subsequent development of epilepsy. In this step, 4 types of neonatal disorders, including neonatal preterm birth, neonatal encephalopathy due to birth asphyxia and trauma, neonatal sepsis and other neonatal infections, hemolytic disease and other neonatal jaundice were identified to be associated with epilepsy. Second, through meta-analysis and other statistical methods, the relative risk (RR) or odds ratio (OR) of injury attributable to neonatal disorders were summarized from the literature. This indicates how many times higher the risk of epilepsy is for those exposed to the risk factor compared to those who are not. Third, GBD model estimated Exposure Rate (P_e) through summarizing global epidemiological data. Here, "exposure" refers to "having survived a severe neonatal disease." Fourth, population attributable fraction (PAF) was

calculated by

PAF = [P_e * (RR - 1)] / [1 + P_e * (RR - 1)] .

PAF means the proportion of all epilepsy cases can be attributed to (are attributable to) past neonatal diseases. In the end, the burden of epilepsy attributable to neonatal diseases can be calculated.

Epilepsy cases attributed to neonatal diseases = Total epilepsy cases × PAF

Epilepsy YLD attributable to neonatal diseases = Total epilepsy YLD × PAF

In summary, GBD quantitatively links epilepsy to neonatal disorders through the bridge of PAF, thereby calculating its prevalence and YLD. To be noticed, the calculation method above is a highly simplified explanation of the actual calculation method used in GBD 2021, as the actual modeling approach is far more complex. Epilepsy has multiple causes (brain injury, stroke, genetics, etc.). GBD calculates the PAF for all causes simultaneously and ensures their sum does not exceed 100%. In addition, with advances in neonatal intensive care technology, more infants with severe neonatal disorders are surviving (changes in P_e), but the subsequent risk of disability (RR) may be altered through interventions. Furthermore, The GBD model captures these temporal trends. All calculations were performed separately by age, sex, country, and year. The P_e of a country with high neonatal mortality is completely different from that of a high-income country, resulting in vastly different attributable burdens

4. The procedure for extracting data about epilepsy attributable to neonatal disorders.

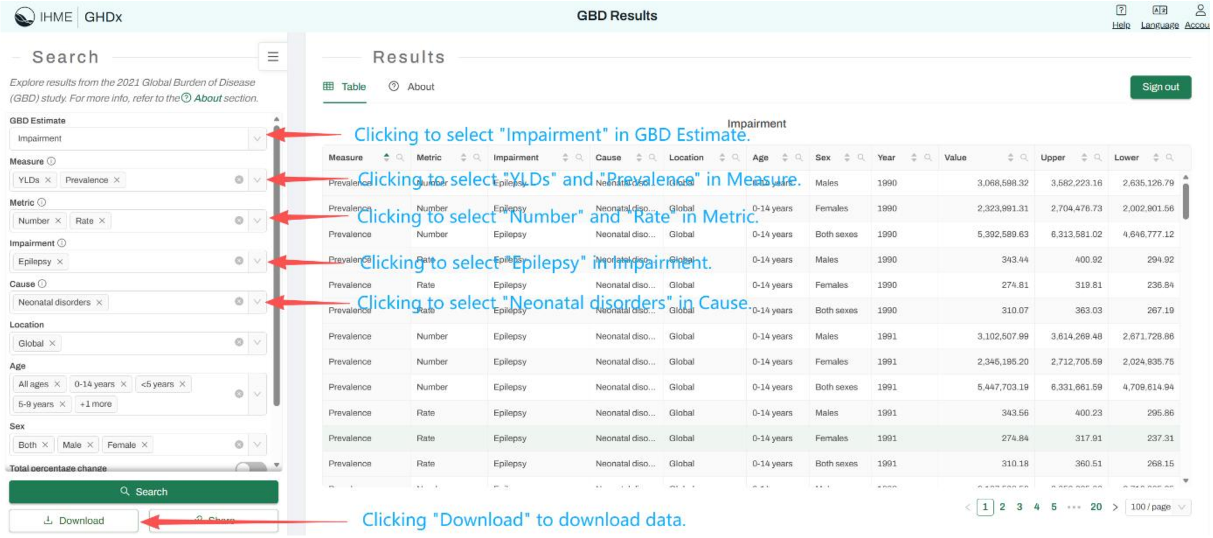

5. Analytic workflow of our study.

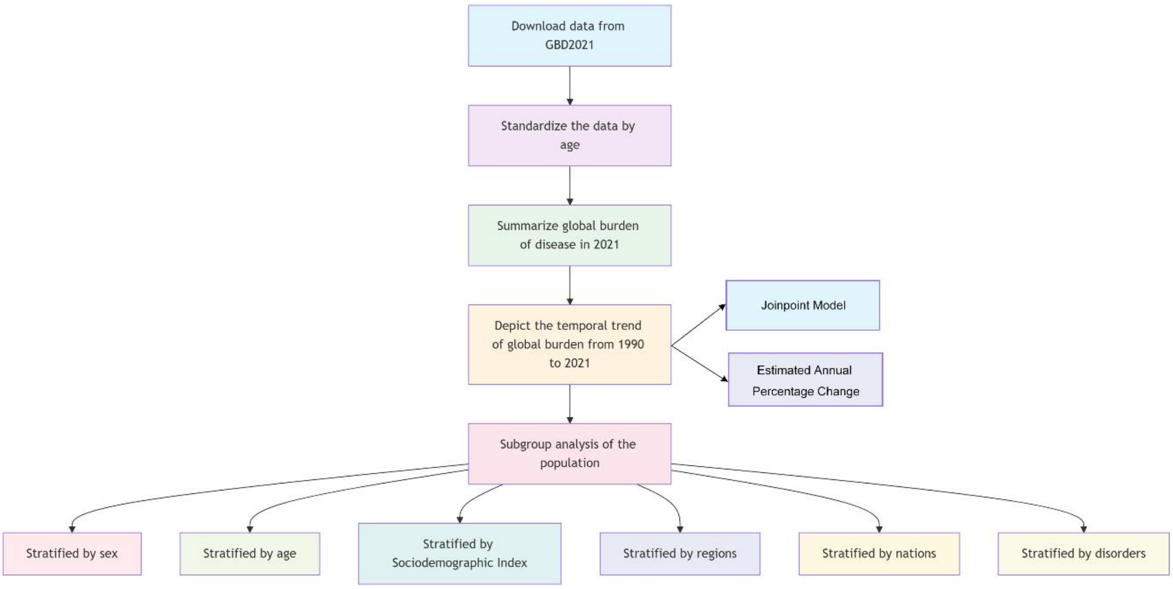

Supplementary Tables

Supplementary Table 1: Age-standardized YLDs rate and YLDs number of epilepsy attributable to neonatal disorders in children under 15 years and their EAPCs from 1990 to 2021 at the global and SDI levels.

	1990		2021		1990-2021
**Characters**	**YLDs number(thousands) (95%UI)**	**Age-standardized YLDs rates (95%UI)**	**YLDs number(thousands) (95%UI)**	**Age-standardized YLDs rates (95%UI)**	**EAPC (95%CI)**
**Global**	1973 (1273.7 to 2745)	113.29 (72.91 to 158.81)	2325 (1526.8 to 3225.1)	115.58 (75.65 to 161.52)	0.1 (0.06 to 0.14)
**Sex**					
**Male**	1124.4 (719.7 to 1568)	125.65 (80.43 to 176.5)	1284.2 (842.6 to 1790.8)	123.66 (80.79 to 174.17)	-0.03 (-0.07 to 0.01)
**Female**	848.6 (548.1 to 1176.3)	100.23 (64.48 to 140.43)	1040.8 (694.3 to 1428.2)	106.97 (70.19 to 148.89)	0.28 (0.23 to 0.32)
**Both**	1973 (1273.7 to 2745)	113.29 (72.91 to 158.81)	2325 (1526.8 to 3225.1)	115.58 (75.65 to 161.52)	0.1 (0.06 to 0.14)
**Age**					
**<15**	1973 (1273.7 to 2745)	113.29 (72.91 to 158.81)	2325 (1526.8 to 3225.1)	115.58 (75.65 to 161.52)	0.1 (0.06 to 0.14)
**0-4 years**	788.2 (516.2 to 1099.9)	127.15 (83.27 to 177.42)	763.1 (505.3 to 1063.7)	115.9 (76.8 to 161.6)	-0.31 (-0.36 to -0.27)
**5-9 years**	640.9 (407.9 to 892.3)	109.83 (69.91 to 152.91)	794.1 (511.4 to 1098.9)	115.6 (74.4 to 159.9)	0.23 (0.18 to 0.28)
**10-14 years**	543.9 (345.9 to 773.4)	101.54 (64.56 to 144.38)	767.8 (504.6 to 1087.2)	115.2 (75.7 to 163.1)	0.48 (0.44 to 0.53)
**SDI**					
**High**	150.2 (89.2 to 226.2)	80.8 (47.82 to 121.92)	184 (118 to 263.6)	70.75 (41.41 to 112.9)	-0.39 (-0.43 to -0.36)
**High middle**	268.4 (167 to 390.4)	98.15 (61.77 to 142.43)	122.3 (70.9 to 195.5)	79.41 (49.51 to 115.24)	-0.65 (-0.73 to -0.57)
**Middle**	586.5 (368 to 845.2)	101.67 (63.22 to 146.88)	805.5 (518.9 to 1138.4)	104.54 (66.99 to 147.46)	0.19 (0.08 to 0.29)
**Low middle**	710.9 (447 to 1001.9)	149.91 (95.56 to 215.27)	618.2 (398.4 to 850.8)	138.82 (88.86 to 198.54)	-0.25 (-0.3 to -0.2)
**Low**	255.2 (156.5 to 366.1)	108.99 (66.74 to 158.78)	593.1 (382.4 to 828.4)	134.25 (86.93 to 187.82)	0.75 (0.64 to 0.87)

Note: Estimates are for children under 15 years. EAPCs= estimated annual percent change. UI = uncertainty interval. CI=confidence interval. YLDs: years lived with disability.

Supplementary Table 2: Age-standardized YLDs rate and YLDs number of epilepsy attributable to neonatal disorders in children under 15 years and their EAPCs from 1990 to 2021 at the regions levels.

	1990		2021		1990-2021
**Characters**	**YLDs number(thousands) (95%UI)**	**Age-standardized YLDs rates (95%UI)**	**YLDs number(thousands) (95%UI)**	**Age-standardized YLDs rates (95%UI)**	**EAPC (95%CI)**
**Global**	1973 (1273.7 to 2745)	113.29 (72.91 to 158.81)	2325 (1526.8 to 3225.1)	115.58 (75.65 to 161.52)	0.1 (0.06 to 0.14)
**Region**					
**High-income Asia Pacific**	23.3 (13.3 to 35.4)	66.38 (37.91 to 101.66)	12.1 (6.6 to 19.7)	53.92 (29.56 to 88.28)	-0.6 (-0.65 to -0.55)
**High-income North America**	52.3 (30.2 to 82)	84.8 (48.4 to 133.22)	50.6 (28.1 to 81)	76.91 (42.59 to 124.38)	-0.34 (-0.42 to -0.26)
**Western Europe**	51.1 (29.1 to 81.5)	72 (40.95 to 116.01)	42.2 (22.7 to 70.9)	61.93 (33.25 to 104.27)	-0.42 (-0.45 to -0.39)
**Australasia**	3.9 (1.8 to 7.3)	85.28 (39.78 to 158.09)	3.6 (1.6 to 7.1)	62.49 (26.9 to 123.1)	-1.04 (-1.09 to -0.99)
**Andean Latin America**	17.6 (10.3 to 27)	118.64 (69.19 to 183.51)	18.9 (10.7 to 29)	104.45 (58.88 to 161.84)	-0.23 (-0.32 to -0.14)
**Tropical Latin America**	43.1 (26.6 to 64.2)	80.91 (50.21 to 120.25)	59.3 (37.8 to 85.9)	118.16 (74.84 to 171.11)	1.56 (1.44 to 1.68)
**Central Latin America**	78.5 (48 to 117.6)	121.77 (74.82 to 183.67)	77 (49 to 111.1)	121.48 (76.9 to 176.09)	0.03 (0.01 to 0.05)
**Southern Latin America**	16 (9.6 to 25.1)	107.32 (63.37 to 167.03)	14.8 (7.4 to 25.4)	101.38 (50.35 to 173.42)	-0.12 (-0.14 to -0.09)
**Caribbean**	19.9 (12.7 to 27.4)	173.98 (109.98 to 242.84)	17.7 (11.6 to 25)	154.09 (97.73 to 223.26)	-0.31 (-0.38 to -0.24)
**Central Europe**	34.8 (22.4 to 51.1)	117.15 (73.56 to 174.87)	16.5 (10 to 25.2)	92.5 (54.37 to 142.9)	-0.76 (-0.79 to -0.74)
**Eastern Europe**	48.5 (29.4 to 71.8)	93.87 (56.24 to 142.8)	27.6 (16.6 to 41.6)	76.45 (44.6 to 117.74)	-0.81 (-0.92 to -0.7)
**Central Asia**	25.3 (15.8 to 38.3)	101.01 (62.69 to 151.79)	30.7 (18.4 to 45.2)	110.92 (66.71 to 163.27)	0.35 (0.28 to 0.41)
**North Africa and Middle East**	193.4 (117.9 to 278.3)	137.25 (83.69 to 198)	225.5 (145.7 to 326.4)	123.07 (79.2 to 178.09)	-0.26 (-0.3 to -0.22)
**South Asia**	797 (503.2 to 1124.6)	183.28 (114.92 to 266.42)	721.5 (451.2 to 1054.2)	141.94 (86.58 to 214.72)	-0.87 (-1 to -0.73)
**Southeast Asia**	179.7 (110.6 to 257.1)	105.27 (64.65 to 152.03)	204.7 (133.1 to 281.5)	118.45 (76.57 to 163.15)	0.42 (0.35 to 0.5)
**East Asia**	227.5 (138.4 to 344.8)	68.99 (41.56 to 105.4)	173.1 (106.6 to 251.7)	64.67 (39.61 to 94.21)	-0.03 (-0.17 to 0.11)
**Oceania**	3.5 (2.2 to 5)	129.85 (80.77 to 191.38)	6.4 (3.9 to 9.5)	125.96 (75.58 to 187.88)	-0.07 (-0.12 to -0.01)
**Western**	50.3 (30.1 to 75)	54.5 (32.45 to 81.98	233.6 (148.1 to	108 (68.17 to 149.0	2.44

Characters	1990 YLDs number(thousands) (95%UI)	1990 Age-standardized YLDs rates (95%UI)	2021 YLDs number(thousands) (95%UI)	2021 Age-standardized YLDs rates (95%UI)	1990-2021 EAPC (95%CI)
**Sub-Saharan Africa**		)	319.9)	8)	(2.1 to 2.79)
**Eastern Sub-Saharan Africa**	67 (39.4 to 101.3)	71.75 (41.74 to 108. 51)	279.3 (182.2 to 390.7)	156.67 (99.68 to 22 0.74)	3.03 (2.85 to 3.2 1)
**Central Sub-Saharan Africa**	16.9 (9.2 to 26.1)	63.39 (34.54 to 97.8 7)	71.6 (42.1 to 106.9)	121.73 (71.93 to 18 2.91)	2.58 (2.16 to 3)
**Southern Sub-Saharan Africa**	23.4 (14.1 to 35.5)	113.04 (67.24 to 172 .95)	38.3 (23.9 to 53.3)	158.96 (96.56 to 22 4.71)	1.23 (1.06 to 1.4)

Note: Estimates are for children under 15 years. EAPCs= estimated annual percent change. UI = uncertainty interval. CI=confidence interval. YLDs: years lived with disability.

Supplementary Table 3:

See Supplementary Table 3 in the separate Excel file for details.

Supplementary Table 4:

See Supplementary Table 4 in the separate Excel file for details.

Supplementary Table 5: Age-standardized rate of YLDs and prevalence of epilepsy attributable to four types of neonatal disorders in children under 15 years in population subgroups.

Measure	Causes	All	Male	Female	<5	5 to 9	10 to 14	Low SDI	Low-middle SDI	Middle-SDI	High-middle SDI	High SDI
ASPR	Neonatal preterm birth	218.16 (179.2 to 260.86)	226.43 (183.81 to 272.79)	209.34 (173.67 to 248.31)	219.73 (184.91 to 260.03)	215.93 (176.87 to 255.04)	218.8 (175.31 to 268.02)	205.12 (167.88 to 247.26)	286.24 (225.42 to 361.32)	193.82 (160.18 to 229.27)	145.61 (124.51 to 166.39)	198.39 (226.5 to 173.29)
ASPR	Neonatal encephalopathy due to birth asphyxia and trauma	79.55 (64.38 to 96.48)	91.53 (74.08 to 110.41)	66.78 (53.93 to 81.92)	76.82 (62.28 to 94.29)	82.21 (66.69 to 99.04)	79.75 (64.27 to 96.17)	85.69 (62.36 to 113.7)	58.93 (44.92 to 74.57)	95.11 (79.82 to 112.07)	92.52 (78.52 to 107.83)	63.72 (71.9 to 56.16)
ASPR	Neonatal sepsis and other neonatal infections	46.98 (30.79 to 66.4)	52.97 (34.54 to 74.88)	40.59 (26.62 to 57.65)	45.73 (30.29 to 64.86)	48 (31.66 to 67.55)	47.28 (30.4 to 66.89)	53.91 (34.78 to 77)	46.65 (30.21 to 66.91)	43.47 (28.06 to 61.77)	50.59 (33.21 to 70.34)	35.88 (50.83 to 23.02)
ASPR	Hemolytic disease and other neonatal jaundice	14.02 (11.79 to 16.34)	13.81 (11.53 to 16.17)	14.25 (12.08 to 16.55)	14.33 (12.05 to 16.89)	14.02 (11.93 to 16.18)	13.68 (11.37 to 15.91)	17.15 (14.47 to 20.04)	15.82 (13.04 to 18.79)	14.28 (12.25 to 16.53)	10.27 (8.46 to 12.11)	3.58 (4.19 to 3.09)
Age-standardized YLDs rate	Neonatal preterm birth	70.44 (45.34 to 100.88)	73.11 (46.96 to 105.37)	67.6 (43.87 to 96.19)	71.56 (46.29 to 101.01)	69.4 (45.08 to 100.16)	70.31 (44.57 to 101.51)	75.38 (47.92 to 108.34)	97.22 (61.42 to 143.72)	58.75 (37.68 to 84.51)	39.05 (23.81 to 58.4)	46.51 (26.9 to 75.66)
Age-standardized YLDs rate	Neonatal encephalopathy due to birth asphyxia and trauma	25.2 (16.12 to 36.95)	28.75 (18.4 to 42.27)	21.42 (13.67 to 31.38)	24.56 (16.13 to 35.99)	25.97 (16.26 to 38.09)	25.09 (15.96 to 36.8)	32.21 (18.99 to 48.99)	20.15 (12.54 to 30.17)	28.14 (18.11 to 41.32)	24.22 (14.86 to 36.36)	14.98 (8.6 to 23.69)
Age-standardized YLDs rate	Neonatal sepsis and other neonatal infections	15.25 (8.34 to 23.61)	17.2 (9.34 to 26.61)	13.18 (7.21 to 20.35)	14.98 (8.14 to 23.22)	15.54 (8.56 to 23.87)	15.24 (8.34 to 23.77)	20.13 (10.87 to 31.55)	16.01 (8.64 to 25.34)	13.33 (7.21 to 20.78)	13.36 (6.67 to 21.46)	8.38 (3.97 to 13.89)
Age-standardized YLDs rate	Hemolytic disease and other neonatal jaundice	4.69 (3.13 to 6.52)	4.6 (3.08 to 6.43)	4.78 (3.21 to 6.64)	4.84 (3.3 to 6.7)	4.67 (3.06 to 6.48)	4.54 (3.01 to 6.35)	6.53 (4.32 to 9.24)	5.44 (3.56 to 7.7)	4.32 (2.84 to 6.13)	2.77 (1.73 to 4.2)	0.88 (0.51 to 1.39)

Note: Estimates are for children under 15 years. UI = uncertainty interval. ASPR: age-standardized prevalence rate. YLDs: years lived with disability.

Supplementary Table 6: EAPC of age-standardized rate of YLDs and prevalence of epilepsy attributable to four types of neonatal disorders in children under 15 years in population subgroups.

**Measure**	**Causes**	**All (95% CI)**	**Male (95% CI)**	**Female (95% CI)**	**<5 years (95% CI)**	**5 to 9 years (95% CI)**	**10 to 14 years (95% CI)**	**Low SDI (95% CI)**	**Low-middle SDI (95% CI)**	**Middle SDI (95% CI)**	**High-middle SDI (95% CI)**	**High SDI (95% CI)**
**EAPC of ASPR**	Neonatal encephalopathy due to birth asphyxia and trauma	1.92 (1.64 to 2.2)	1.8 (1.56 to 2.04)	2.09 (1.73 to 2.45)	1.62(1.33 to 1.92)	2.11(1.78 to 2.45)	2.05(1.75 to 2.34)	6.06 (4.97 to 7.16)	3.45 (3.37 to 3.54)	1.75 (1.16 to 2.34)	0.91 (0.59 to 1.22)	0.04 (-0.03 to 0.1)
**EAPC of ASPR**	Neonatal sepsis and other neonatal infections	1.11 (0.9 to 1.33)	1.14 (0.87 to 1.41)	1.07 (0.95 to 1.19)	0.73(0.42 to 1.04)	1.31(1.12 to 1.51)	1.36(1.13 to 1.59)	4.06 (3.58 to 4.53)	2.08 (1.66 to 2.5)	1.09 (0.09 to 2.1)	-0.55 (-1.35 to 0.25)	-0.42 (-1.02 to 0.18)
**EAPC of ASPR**	Hemolytic disease and other neonatal jaundice	0.5 (0.31 to 0.69)	0.47 (0.3 to 0.64)	0.53 (0.31 to 0.76)	0.38(0.16 to 0.59)	0.52(0.32 to 0.73)	0.62(0.44 to 0.81)	0.16 (0.1 to 0.21)	0.2 (0.01 to 0.38)	0.66 (0.44 to 0.88)	0.07 (-0.05 to 0.19)	0.2 (0.09 to 0.31)
**EAPC of ASPR**	Neonatal preterm birth	0 (-0.32 to 0.33)	-0.17 (-0.5 to 0.16)	0.22 (-0.11 to 0.55)	-0.36(-0.57 to -0.14)	0.06(-0.32 to 0.45)	0.39(-0.03 to 0.82)	-0.12 (-0.36 to 0.13)	-0.28 (-0.59 to 0.02)	0.28 (-0.09 to 0.65)	-0.23 (-0.38 to -0.09)	0.05 (-0.13 to 0.23)
**EAPC of age-standardized YLDs rate**	Neonatal encephalopathy due to birth asphyxia and trauma	1.64(1.27 to 2)	1.5(1.18 to 1.83)	1.83(1.39 to 2.27)	1.31(0.96 to 1.67)	1.84(1.43 to 2.26)	1.8(1.42 to 2.18)	5.66(4.63 to 6.7)	3(2.9 to 3.1)	1.09(0.52 to 1.66)	0.17(-0.04 to 0.39)	-0.39(-0.54 to -0.24)
**EAPC of age-standardized YLDs rate**	Neonatal sepsis and other neonatal infections	0.9(0.7 to 1.1)	0.91(0.65 to 1.16)	0.88(0.76 to 1.01)	0.46(0.18 to 0.74)	1.12(0.92 to 1.32)	1.2(0.96 to 1.44)	3.66(3.19 to 4.13)	1.62(1.18 to 2.06)	0.56(-0.41 to 1.54)	-1.14(-1.93 to -0.35)	-0.89(-1.35 to -0.43)
**EAPC of age-standardized YLDs rate**	Hemolytic disease and other neonatal jaundice	0.07(-0.17 to 0.31)	0.04(-0.17 to 0.25)	0.11(-0.17 to 0.39)	-0.06(-0.33 to 0.22)	0.09(-0.16 to 0.35)	0.2(-0.02 to 0.43)	-0.2(-0.31 to -0.1)	-0.32(-0.57 to -0.07)	0.05(-0.15 to 0.26)	-0.55(-0.65 to -0.45)	-0.31(-0.37 to -0.24)

**EAPC of age-standardized YLDs rate**	Neonatal preterm birth	-0.46(-0.82 to -0.1)	-0.65(-1.01 to -0.28)	-0.23(-0.6 to 0.13)	-0.83(-1.09 to -0.57)	-0.41(-0.84 to 0.02)	-0.05(-0.5 to 0.41)	-0.51(-0.73 to -0.3)	-0.82(-1.19 to -0.45)	-0.33(-0.7 to 0.04)	-0.95(-1.06 to -0.85)	-0.34(-0.35 to -0.33)

Note: Estimates are for children under 15 years. EAPCs= estimated annual percent change. CI=confidence interval. ASPR: age-standardized prevalence rate. YLDs: years lived with disability.

Supplementary Table 7: Number of YLDs and prevalence of epilepsy attributable to four types of neonatal disorders in children under 15 years in population subgroups in 2021.

measure_	location	sex	age	cause	rei	metric	year	val	upper	lower	proportion
**Prevalence**	Global	Both	0-14 years	Neonatal preterm birth	Epilepsy	Number	2021	4388365	5114647	3699273	0.607959
**Prevalence**	Global	Both	0-14 years	Neonatal encephalopathy due to birth asphyxia and trauma	Epilepsy	Number	2021	1602063	1934488	1316416	0.221948
**Prevalence**	Global	Both	0-14 years	Neonatal sepsis and other neonatal infections	Epilepsy	Number	2021	945903.6	1342850	621914.3	0.131044
**Prevalence**	Global	Both	0-14 years	Hemolytic disease and other neonatal jaundice	Epilepsy	Number	2021	281858.2	325827.7	240038.9	0.039048
**Prevalence**	Global	Both	0-14 years	Neonatal disorders	Epilepsy	Number	2021	7218190	8112952	6296280	1
**YLDs (Years Lived with Disability)**	Global	Both	0-14 years	Neonatal preterm birth	Epilepsy	Number	2021	1416559	2022071	935365.2	0.609265
**YLDs (Years Lived with Disability)**	Global	Both	0-14 years	Neonatal encephalopathy due to birth asphyxia and trauma	Epilepsy	Number	2021	507316.2	738331.6	328856.1	0.218198
**YLDs (Years Lived with Disability)**	Global	Both	0-14 years	Neonatal sepsis and other neonatal infections	Epilepsy	Number	2021	306991.6	478292.3	170155.4	0.132038
**YLDs (Years Lived with Disability)**	Global	Both	0-14 years	Hemolytic disease and other neonatal jaundice	Epilepsy	Number	2021	94161.82	129871.6	62676.19	0.040499
**YLDs (Years Lived with Disability)**	Global	Both	0-14 years	Neonatal disorders	Epilepsy	Number	2021	2325029	3225095	1526829	1

Note: Estimates are for children under 15 years. YLDs: years lived with disability.
